# An interacting quantum atom study of model S_N_2 reactions (X^–^···CH_3_X, X = F, Cl, Br, and I)

**DOI:** 10.1002/jcc.25098

**Published:** 2017-11-10

**Authors:** Ibon Alkorta, Joseph C. R. Thacker, Paul L. A. Popelier

**Affiliations:** ^1^ Instituto de Química Médica (CSIC), Juan de la Cierva, 3 Madrid 28006 Spain; ^2^ Manchester Institute of Biotechnology (MIB), 131 Princess Street, M1 7DN, Great Britain, and School of Chemistry, University of Manchester, Oxford Road Manchester M13 9PL Great Britain

**Keywords:** quantum chemical topology, interacting quantum atoms, relative energy gradient, S_N_2

## Abstract

The quantum chemical topology method has been used to analyze the energetic profiles in the X^–^ + CH_3_X → XCH_3_ + X^–^S_N_2 reactions, with X = F, Cl, Br, and I. The evolution of the electron density properties at the BCPs along the reaction coordinate has been analysed. The interacting quantum atoms (IQA) method has been used to evaluate the intra‐atomic and interatomic energy variations along the reaction path. The different energetic terms have been examined by the relative energy gradient method and the *ANANKE* program, which enables automatic and unbiased IQA analysis. Four of the six most important IQA energy contributions were needed to reproduce the reaction barrier common to all reactions. The four reactions considered share many common characteristics but when X = F a number of particularities occur. © 2017 Wiley Periodicals, Inc.

## Introduction

Understanding the forces that act on atoms in reaction processes is an important challenge in modern chemistry that remains currently unsolved. Any progress on this matter can have profound implications for a number of fields, from biochemistry to industrial catalysis. The S_N_2 reaction is a type of reaction mechanism that is common in organic chemistry, and which can hence serve as an important case study for approaches that offer insight in the energetic composition of reaction profiles. In this mechanism, one bond is broken and one bond is formed in a single step.

For reasons to be described just below, quantum chemical topology (QCT)^1^, ^2^, ^3^, ^4^ is an appealing approach to study the ubiquitous S_N_2 reaction at atomistic level. In this article we use two specific approaches that QCT encompasses, called the quantum theory of atoms in molecules (QTAIM)^1^, ^5^, ^6^ and the interacting quantum atoms (IQA).^7^ Both approaches share the same pivotal idea that a (gradient) vector field partitions^8^ a system at hand, and thereby provides various properties of subspaces, which in the case of QTAIM and IQA are topological atoms. These atoms are space‐filling (i.e., the atoms do not overlap and each point in space belongs to an atom) and are obtained without using either parameters or a reference state (e.g., a promolecule).

Amongst the various energy decomposition analysis (EDA) schemes in existence, IQA presents itself as a modern alternative offering a growing number of applications. Following the EDA acronym tradition, IQA could be referred to as QCTEDA but this name is hard to pronounce. Two popular EDA schemes, but much older than IQA, are derived from a variational method going back to the work of Morokuma,^9^ or constitute the symmetry adapted perturbation method.^10^ A very recent review^11^ on traditional (i.e., non‐QCT) EDA schemes highlights and discusses a number of their typical problems, amongst which there are (i) the lack of a clear chemical significance of its energy components, which are unfortunately not uniquely defined; (ii) the increasingly flawed separation of charge transfer and polarization at close intermolecular distances and with large basis sets (possibly causing numerical instabilities); and (iii) the fact that the mixing term can become greater in magnitude than the total interaction energy itself. IQA does not suffer from any of these issues but suffers from high(er) computational cost. Furthermore, energy partitioning schemes remain inherently arbitrary since no experiment can settle their veracity.

The IQA methodology has been applied, with success, to a growing number of chemical problems, such as hydrogen bonds and cooperativity,^12^, ^13^ metal carbonyl bonds,^14^ bond formation,^15^ halogen bonds,^16^ Zn‐complexes,^17^ analysis of the biphenyl rotational barrier,^18^ excited states,^19^ congested molecules,^20^ intramolecular interactions in substituted trinitromethanes,^21^ protonation,^22^ I_2_ interaction with organoselenium compounds^23^ and tautomerization processes,^24^ conformational analysis of diheteroaryl ketones and thioketones,^25^ diastereoselective allylation of aldehydes,^26^ dissociation profiles,^27^ addition of water to SO_3_,^28^ and CO_2_ trapping by carbenes,^29^ to mention a nonexhaustive list of case studies.

Due its fundamental importance, the S_N_2 reaction has been widely studied experimentally^30^ and computationally. The S_N_2 reaction profile is characterized by two ion‐molecule energy minima: one occurring before the transition state (TS), and one after, while progressing along the reaction coordinate.^31^ Several recent reviews on S_N_2 reactions are available.^32^, ^33^ The complexation energies and S_N_2 reactions of X^–^ + CH_3_X systems (where X = F, Cl, Br, and I) have previously been calculated^34^ at the G2(+) level of theory. The central barriers of these four reactions are within an energetic range of 12.7 kJ mol^−1^, and the sizes of the energetic barriers are ranked as follows: Cl > F > Br > I. Good correlations between the central barrier and the ionization energy of the attacking anion were found, and also between the complexation energy and the Mulliken electronegativities of the halogen atoms. The same authors further explored the mechanism with retention and inversion of configuration.^35^ Several levels of theory were compared to CCSD(T) results on S_N_2 reactions.^36^ Conceptual DFT (at B3LYP/6–311++G(d,p) level) has been used to analyze^37^ reactions involving ClCH_3_Cl and FCH_3_F. Polarization effects and through‐bond interaction were found to dominate in the ClCH_3_Cl reaction, while intermolecular charge transfer dominated in the FCH_3_F reaction. The same authors also explored two different mechanisms in the S_N_2 reaction^38^ OH^–^ + CH_3_F → CH_3_OH + F^–^. The electronic transfer contribution of the reaction flux was found to play a crucial role in its profile. The steric, quantum and electrostatic effects in 59 S_N_2 reactions with general formula XCR^1^R^2^R^3^+ X^–^ (with X = F and Cl) have also been studied.^39^ The steric effect (calculated using the NBO method) dominated the TS barrier but was largely compensated by the exchange‐correlation interactions. The electrostatic effect was linearly correlated with the S_N_2 barrier. The Cl^–^···CH_3_Cl reaction has been studied^40^, ^41^, ^42^ several times due to the availability of experimental data for comparison. The static intrinsic reaction coordinate (IRC) and dynamic simulation of this system has been considered and their associated QTAIM charges analyzed.^43^ The noncovalent index (NCI) was also used to illustrate this S_N_2 reaction in solution.^44^ S_N_2 reactions in systems with different incoming and outgoing halogen atoms, and alternative mechanisms (front‐side attack, double inversion, and hydrogen abstraction) have also been investigated.^45^, ^46^, ^47^, ^48^

The activation strain model^49^, ^50^, ^51^ that divides the relative energy along the reaction path as a sum of the variation of the strain energy within the fragments and the change in the interaction energy of those fragments has been used to analyze the S_N_2 reactions occurring in X^–^ + CH_3_Y systems (where X, Y = F, Cl, Br, and I).^52^ Based on this model, the nucleophilicity is determined by the electron‐donor capability of the nucleophile and leaving‐group ability, which is derived directly from carbon‐leaving group (C—Y) bond strength.^52^ The same model has been used to analyze the competition between E2 elimination and S_N_2 substitution,^53^, ^54^ S_N_2 complexation with transition metals as alternative to oxidation^55^, ^56^ and the effect of replacing the central carbon atom by other one (Si,^57^ P,^58^ N,^59^ O,^59^ F,^59^ and Group 14 Atoms^60^). The effect of bulky substituents at carbon in the S_N_2 reaction has been discussed in the literature. Some authors found that the TS reduces the steric repulsion,^61^, ^62^ while other authors reach the opposite conclusion.^63^

In the present article, QCT has been applied (via QTAIM and IQA) to the model chemical reaction of the second‐order nucleophilic substitution (S_N_2). The systems chosen are X^–^···CH_3_X with X = F, Cl, Br, and I, which provide degenerate reactant/product states. IQA offers *all* possible interatomic energy contributions (as well as intra‐atomic contributions) leading to an abundance of energy terms, even for the small reaction systems studied here, especially because of the further breakdown into electrostatic, exchange‐correlation energy types (also covering kinetic energy in the intra‐atomic energy contributions). The abundance of energy terms (resolved both by locality and physical type) calls for a systematic procedure for the relative importance of each term in the global process, which has very recently been proposed and is called the relative energy gradient (REG) method.^64^ The program ANANKE, an in‐house implementation of the REG method, then offers unbiased and automated chemical insight into S_N_2 reactions.

## Computational Methods

### *Ab initio* calculations: Reaction profiles

The stationary points in the reactions have been located using the M06‐2X DFT functional^65^ and the 6–311++G(d,p) basis set^66^ for all atoms apart from iodine, where we used the 6–311G(d,p) basis set supplemented with *s* and *d* diffuse functions as well as *d* and *f* functions proposed by Radom et al.^67^ Frequency calculations have been carried out at the same computational level to confirm that the structures obtained indeed correspond to true energetic minima and transition states. The minimum energy paths connecting the stationary points (minima and TS) have been calculated using the IRC methodology.^68^ Due to the symmetry of the reaction, only one half of the potential energy surface needed to be calculated (between one of the minima and the TS). In the S_N_2 reactions X^–^···CH_3_X, the number of geometries provided by the IRC scan was, respectively, 60, 73, 89, and 105, for X = F, Cl, Br, and I. All calculations were done using the GAUSSIAN09 program.^69^

### Quantum topological analysis: QTAIM and IQA

The electron density of all the geometries provided by the IRC scan has been analyzed according to QTAIM,^1^, ^70^ which provided the so‐called local properties (i.e., quantum mechanical values evaluated in critical points). The so‐called global properties^71^ (i.e., obtained by integration^72^ over the volume of a topological atom) were obtained by IQA. This approach was inspired by one of the first successful attempts^73^ by the current group to calculate the (Coulomb) potential energy between topological atoms, which was simultaneously achieved^74^ by Salvador et al. This algorithmic‐mathematical advance consisted of a six‐dimensional integration, carried out simultaneously over the two three‐dimensional volumes of the topological atoms involved, and made the formerly mandatory use of the virial theorem^75^ obsolete. An important consequence of this relaxation is that the energy of a topological atom can be calculated when present in a molecular system that is not a stationary point.^8^ Thus, with IQA, one can monitor the changes in energy patterns for *any* geometry along the IRC.

Because IQA has been reviewed so many times we explain only the essence below, and ensure the notation used throughout is clear. The IQA approach partitions the total energy of a system into intra‐atomic and interatomic energy contributions, or

(1) $$E_{\text{total}}=\sum_{A}E_{\text{IQA}}^{A}=\sum_{A}\left(E_{\text{intra}}^{A}+\frac{1}{2}\sum_{B\neq A}V_{\text{inter}}^{AB}\right)=\sum_{A}\left(E_{\text{intra}}^{A}+V_{\text{inter}}^{A}\right)$$

where *A* and *B* represent all atoms in the system. The total energy of the system is recovered when all energetic components are summed over the atoms present. These and other IQA terms were calculated by the AIMAll program,^76^ which for the first time allowed IQA to be compatible with a wave function obtained using a DFT functional, according to a procedure that is carefully explained elsewhere.^77^ This procedure is based on a choice for which there is an alternative option.^78^ The compatibility between IQA and DFT ensures that the sum of all IQA energy contributions is identical to the original (unpartitioned) total energy of the system, except for a numerical error caused by the atomic integration algorithm. In the current work, the largest difference, found in any of the systems and configurations, between this sum and the (original) total energy, was only 2.0 kJ/mol. This residual error is acceptable for the conclusions we want to draw from our data.

The quantity $E_{\text{IQA}}^{A}$ is atom *A*'s energy, combining its internal energy $E_{\text{intra}}^{A}$ with the interaction energy between it and its complete atomic environment. The intra‐atomic energy can be further partitioned as

(2) $$E_{\text{intra}}^{A}=T^{A}+V_{ee}^{AA}+V_{en}^{AA}$$

where *T^A^* represents the kinetic energy of the electrons inside the volume of atom *A,* $V_{ee}^{AA}$ is the intra‐atomic electron–electron potential energy, and $V_{en}^{AA}$ the intra‐atomic electron‐nuclear potential energy. The interatomic energy can be further partitioned,

(3) $$V_{\text{inter}}^{AB}=\left(V_{nn}^{AB}+V_{en}^{AB}+V_{en}^{BA}\right)+V_{ee}^{AB}$$

where the subscript ‘*en*’ represents the electron density of atom *A* interacting with the nucleus of atom *B*. The energy quantity $V_{nn}^{AB}$represents the internuclear repulsion between the nuclei of atoms *A* and *B,* while $V_{ee}^{AB}$ represents the interatomic electron–electron potential energy. The $V_{ee}^{AB}$ energy term can be further partitioned as

(4) $$V_{ee}^{AB}=V_{coul}^{AB}+V_{xc}^{AB}$$

where $V_{coul}^{AB}$ represents the Coulombic energy between the electrons in atoms *A* and *B*, and $V_{xc}^{AB}$ represents the exchange‐correlation interaction between the electrons in atoms *A* and *B*. The interatomic energy can also be written as

(5) $$V_{\text{inter}}^{AB}=V_{cl}^{AB}+V_{xc}^{AB}$$

where the “classical” electrostatic term ( $V_{cl}^{AB}$) is defined as

(6) $$V_{cl}^{AB}=\left(V_{nn}^{AB}+V_{en}^{AB}+V_{ne}^{AB}\right)+V_{coul}^{AB}$$

The exchange‐correlation energy is generally considered to correlate^77^, ^79^ to the degree of covalency appearing in interactions between any two atoms.

### The REG method

The REG method has been developed and explained in great detail in Ref. [ 64]; here, we repeat only the essence. This method needs a sequence of geometries to operate. The sequence may consist of very few snapshots, and is controlled by a control coordinate *s*. In the current study, this control coordinate is the IRC. The REG method essentially contrasts the behavior of the total energy of a system that undergoes a change with that of an (IQA) energy contribution going through the same change. For example, if two water molecules are being pulled apart from their dimeric global energy minimum geometry, an energy profile follows, which can be contrasted with the energy profile of the H…O electrostatic interaction only (i.e., the hydrogen bond itself). The REG method is then interested in how a particular (IQA) energy contribution and the total energy behave relative to each other. More precisely, we calculate the Pearson correlation coefficient *R(X,Y)* between the two energy variables *X* and *Y* (i.e., the particular energy and the total energy), based on the values they adopt for each of the geometries along the IRC.

The REG method owes its name to its derivation through the use of energy gradients. As the IQA energetic partitioning scheme is additive in nature, the total energy of the system is recovered by the following sum,

(7) $$E_{\text{total}}(s)=\sum_{i=1}^{N}E_{i}(s)$$

where the subscript *i* denotes the IQA energy contribution (e.g., $V_{cl}^{CH}$), the subscript *total* refers to the total system, *N* is the total number of energy terms and *s* is the control coordinate, which is sampled at *M* data points (not shown in this equation). On a practical note, we mention that this equation is numerically not exact for the IQA scheme due to its atomic integration errors.^80^

We analyse how the energetically partitioned term changes with respect to the total energy. We relate these two energies using linear regression, as shown in eq. (8),

(8) $$E_{i}(s)=m_{\text{REG},i}\cdot E_{\text{total}}(s)+c_{i}$$

where $m_{\text{REG},i}$ is the REG itself. Note that eq. (8) actually lists an equation for each energy term *i*, which is fitted to the *M* data points. It is, therefore, clear that the REG for a given segment along the IRC can be estimated using the least‐squares linear regression eq. (9),

(9) $$m_{\text{REG},i}=\frac{{\left(E_{\text{total}}^{\text{translated}}\right)}^{\tau }\cdot E_{i}^{\text{translated}}}{{\left(E_{\text{total}}^{\text{translated}}\right)}^{\tau }\cdot E_{\text{total}}^{\text{translated}}}$$

$$\text{where}\;\begin{array}{l} {\left(E_{i}^{\text{translated}}\right)}^{\tau }={\left[\begin{array}{cccc} E_{i}(s_{1})-{\bar{E}}_{i} & E_{i}(s_{2})-{\bar{E}}_{i} & \cdots & E_{i}(s_{M})-{\bar{E}}_{i} \end{array}\right]}^{\tau }\\ {\left(E_{\text{total}}^{\text{translated}}\right)}^{\tau }={\left[\begin{array}{cccc} E_{\text{total}}(s_{1})-{\bar{E}}_{\text{total}} & E_{\text{total}}(s_{2})-{\bar{E}}_{\text{total}} & \cdots & E_{\text{total}}(s_{M})-{\bar{E}}_{\text{total}} \end{array}\right]}^{\tau } \end{array}$$

and the superscript bar represents the average over the *M* data points, while the translation results from the subtraction of the respective averages.

The REG is therefore only valid when there is strong linearity between the total energy and the energetically partitioned term (i.e., the energy contribution). This degree of linearity is assessed using the Pearson correlation coefficient (*R_i_*), as defined in eq. (10) for the *i*th energy contribution,

(10) $$R_{i}=\frac{\sum_{s}^{M}E_{i}^{\text{translated}}{(s)E}_{i}^{\text{total}}(s)}{\sqrt{\sum_{s}^{M}{\left(E_{i}^{\text{translated}}(s)\right)}^{2}}\sqrt{\sum_{s}^{M}{\left(E_{\text{total}}^{\text{translated}}(s)\right)}^{2}}}$$

where *M* is the number of values that the control coordinate adopts.

This method is called the relative‐energy‐gradient method as it indeed compares the *gradient* of a given IQA energy contribution and the gradient of the total system energy. This is clear when the derivative of eq. (8) is taken with respect to *s*, as seen in eq. (11),

(11) $$\frac{dE_{i}(s)}{ds}=m_{\text{REG},i}\cdot \frac{dE_{\text{total}}(s)}{ds}$$

Note that eq. (11) is only valid for perfect correlation, and becomes increasingly approximate as the correlation deteriorates. Indeed, in that case, the ratio of derivatives, obtainable from eq. (11), increasingly deviates from *m*_REG,__*i*_.

It is important to note that REGs ( $m_{\text{REG},i}$) can have both positive and negative values. Positive REG values represent IQA energy contributions that have the same sign in energy gradient as the total system. In other words, the energy gradients associated with these terms have the same sign as the gradient associated with the total system's energy. In contrast, negative REG values have an opposite sign to the energy gradient associated with the total energy. Finally, put differently again and concluding, IQA terms with positive REGs help construct a given energy barrier, working in the same direction as the barrier. Conversely, negative REGs work against the total energy barrier. The program ANANKE will calculate the REG values (i.e., *m*_REG,__*i*_) and rank the IQA energy contributions; the largest REG is the most significant IQA term leading to the observed behavior of the system.

Finally, we point out that the REG method has no dependency on the reaction coordinate itself, so long as all the data originate from the same set of reaction coordinates, and also that the reaction coordinate is well sampled in the region studied. In other words, after the control coordinate has done its task of providing a sequence of geometries it disappears from the calculation of the REGs. In this respect the REG method removes the need to define the reaction coordinate during calculation. This is useful as it allows for the reaction coordinate to be ill‐defined or complicated (as is often the case in chemistry, particularly using an internal reaction coordinate [IRC]).

## Results and Discussion

### Geometries and energies

**Figure 1:**
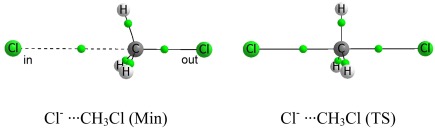
Molecular graph of the stationary points of the Cl^–^···CH_3_Cl system, indicating the incoming (left) and outgoing chlorine (right). The small green spheres represent bond critical points (BCPs), which are explained later in the main text. [Color figure can be viewed at wileyonlinelibrary.com]

The energetic profile of the four X^–^···CH_3_X systems in the gas phase, is characterized by the presence of two nondegenerate stationary points: a minimum and a TS. The minimum possesses C_3v_ symmetry while the TS has D_3h_ symmetry (Fig. 1).

In the minima of the X^–^···CH_3_X systems, the X—C distances are 1.429 Å and 2.529 Å for X = F, 1.828 Å and 3.117 Å for X = Cl, 1.986 Å and 3.253 Å for X = Br, and 2.173 Å and 3.486 Å for X = I. The X—C bond distances in the TS are 1.804 Å, 2.313 Å, 2.462 Å, and 2.660 Å, respectively.

**Figure 2:**
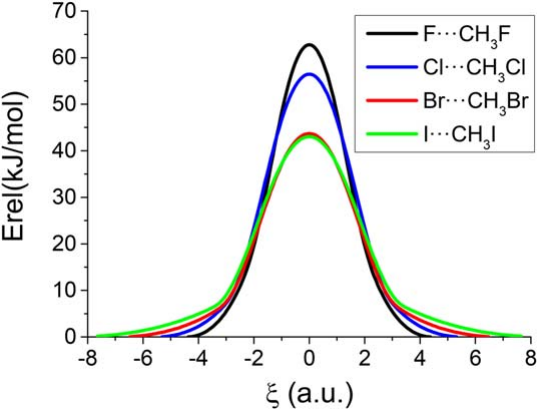
Energetic profile of all four halogen systems (kJ/mol) along the reaction coordinate ξ (a.u.). The energies of the respective minima (one for each halogen‐substituted compound) have been used as references (*E*_min_ = 0.0 kJ/mol). [Color figure can be viewed at wileyonlinelibrary.com]

Figure 2 shows the energetic profiles of all four systems. The calculated barriers between the minimum and the TS for the X^–^···CH_3_X systems decrease as the size of the halogen atom increases, being 62.8, 56.4, 43.7, and 43.0 kJ/mol for X = F, Cl, Br, and I, respectively. The calculated barrier for X = Cl is in good agreement with the experimental^30^ one (55.2 ± 8.4 kJ/mol) and with previous high‐level *ab initio* calculations.^34^, ^36^

### Evolution of the properties at the BCPs along the reaction coordinate

**Figure 3:**
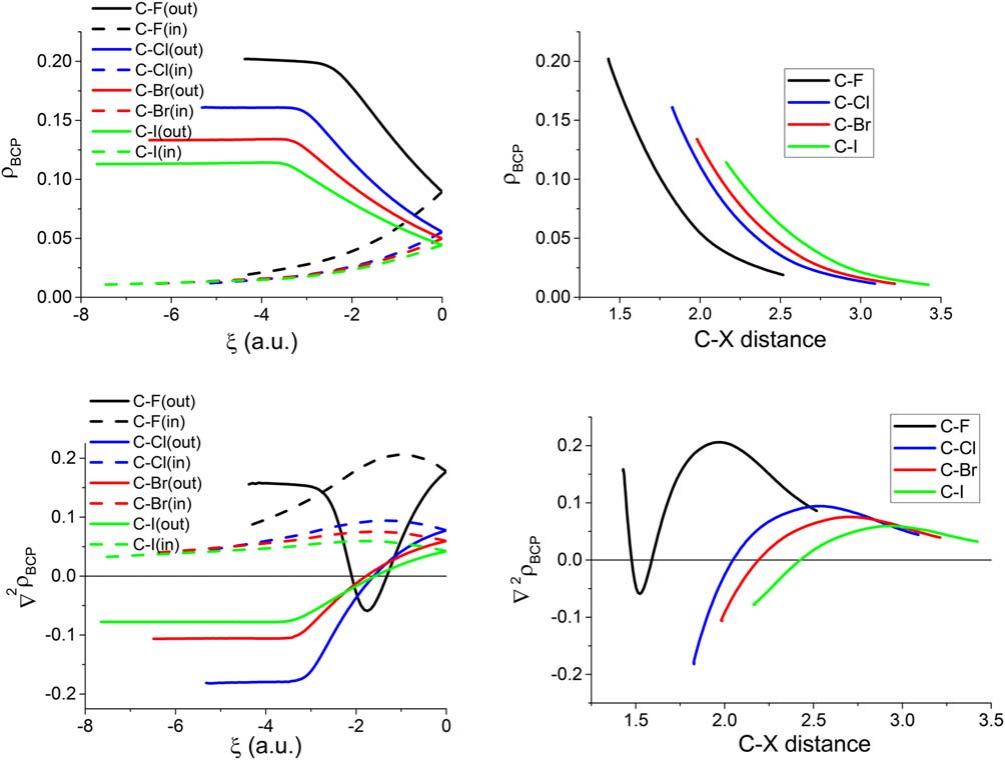
Variation of *ρ*_BCP_ and ∇^2^ *ρ*_BCP_ (a.u.) at the respective C—X BCPs as a function of the reaction coordinate ξ (a.u.) (left panels) and of the C—X distance (Å) (right panels). [Color figure can be viewed at wileyonlinelibrary.com]

Figure 3 shows the evolution of the electron density properties of the C—X bond critical point (BCP) as a function of the reaction coordinate (left panels) and of the interatomic C—X distance (right panels). Loosely speaking, a BCP is a saddle‐type of critical point that marks the topological boundary between two topologically connected atoms (more precise details see Ref. [ 8]). Evaluating properties at BCPs has many applications including in molecular similarity^81^ and quantitative‐structure–activity relationships.^82^ The values of the electron density at the BCP (*ρ*_BCP_) in the initial stage of the reactions remain almost unaltered for the C—*X*_out_ bonds (solid curves in the top left panel of Fig. 3), up to ξ ∼ −3, beyond which point *ρ*_BCP_ decreases rapidly. This pattern of a plateau followed by a sudden drop is not seen in the *ρ*_BCP_ values of C—*X*_in_ bonds (dashed curves in top left panel) where instead, *ρ*_BCP_ values smoothly increase along the reaction path, until they meet the respective *ρ*_BCP_ values of the C—*X*_out_ bonds, at the TS. The evolution of *ρ*_BCP_ as a function of interatomic distance is compatible with known exponential relationships between these two variables.^83^

Note that the C—F bond shows the most dramatic changes compared to the other C—X bonds. This unique behavior of F also occurs in other profiles studied (see below), and is vaguely reminiscent of the unique bonding behavior^84^ of C in its own group (Group 14). The peculiarities of the C—F interactions are well known and attributed by some authors to charge‐shift bonding.^85^ Atoms (or fragments) that are prone to this type of bonding are compact electronegative and/or lone‐pair‐rich species. In more general terms, the phenomenon that second row elements are chemically more unique compared to their third and fourth row counterparts is universal and has been found in other groups of the periodic table.

The variation of the Laplacian at the bond critical point (∇^2^ *ρ*_BCP_) as a function of the reaction coordinate and of the interatomic distance is different in F^–^···CH_3_F compared to the other systems. Again, F sets itself apart from the other halogens. In the minimum conformation, the two C—F bonds show positive values of ∇^2^ *ρ*_BCP_, while the corresponding C—X values are negative. Moving to the right along the reaction coordinate, the ∇^2^ *ρ*_BCP_ values for C—*F*_out_ decrease, starting from the minimum up to ξ = −1.75 a.u., where a deep minimum in ∇^2^ *ρ*_BCP_ occurs. Subsequently, ∇^2^ *ρ*_BCP_ increases until it reaches the positive TS value. In summary, ∇^2^ *ρ*_BCP_(C—*F*_out_) displays a unique profile that is topologically different from any other profile in the bottom left panel of Figure 3. Again moving to the right starting from the left minimum, the Laplacian of C—*F*_in_ increases its value until ξ ∼ −1.0 a.u. and beyond which point it decreases. The other ∇^2^ *ρ*_BCP_ (C—*X*_in_) profiles show a similar behavior but with a less pronounced maximum around ξ = −1.5 a.u. The ∇^2^ *ρ*_BCP_(C—*X*_out_) values for X = Cl, Br, and I, are negative at the energy minimum and remain almost unaltered at the beginning of the reaction until ξ ∼ −3.0 a.u. where they start to increase their values and cross each other's profiles while reaching positive values at ξ = −1.6, approximately.

The behavior of ∇^2^ *ρ*_BCP_ (C—F) as a function of interatomic distance (bottom right panel in Fig. 3) is again different to that of the other C—X bonds, and resembles the profile recently described for the P—N interactions.^86^ The rest of the C—X bonds (X = Cl, Br, and I) shows a similar Laplacian profile: negative for small interatomic distances increasing as the distance increases, until it shows a small positive maximum and later decreases for longer distances toward zero at infinite distance.^87^

### IQA analysis

The IQA analysis will be divided in four parts, each discussed in one of the four subheaders, and starting with the coarsest examination. First the variation of the *total* intra‐atomic and interatomic energies along the reaction coordinate will be considered. In the second part, the atomic contributions to the intra‐atomic and interatomic energies, as well as the QTAIM atomic charges, will be discussed. In the third part, the different terms of the interatomic energies will be examined. In the last part, the REG method analyses of the importance of the different IQA energy contributions using the ANANKE program.

#### Total intra‐atomic and interatomic energies

**Figure 4:**
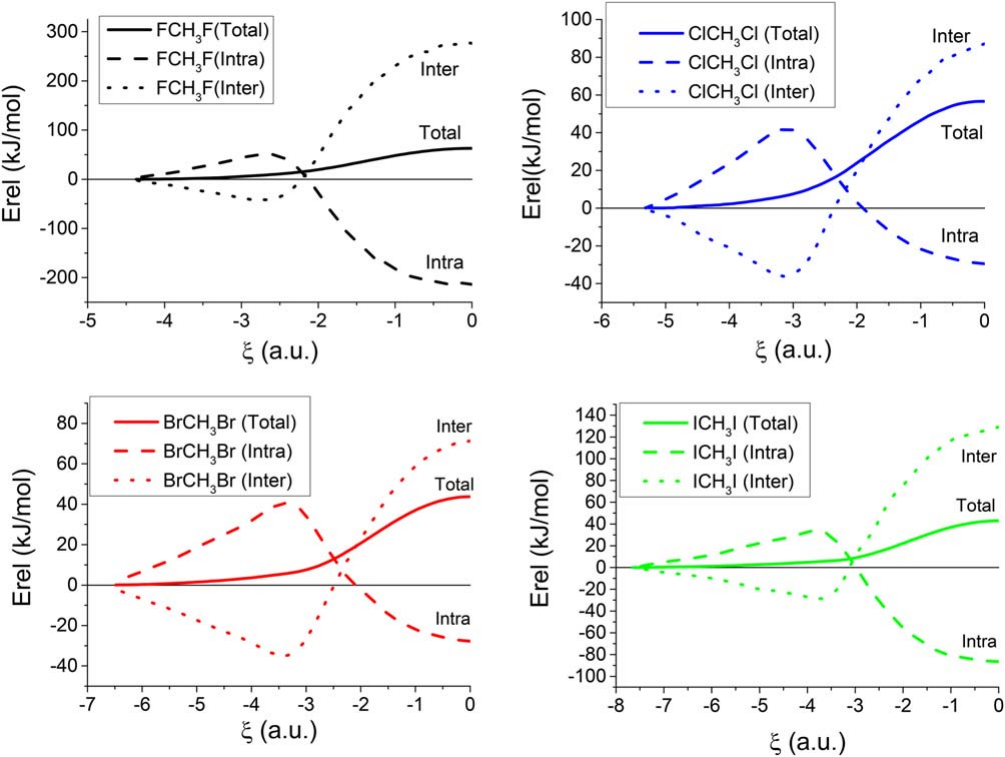
Variation of the total intra‐atomic energy, interatomic energy and total energy in the X^–^···CH_3_X systems (X= F, Cl, Br, and I) *versus* the reaction coordinate ξ, where ξ = 0 corresponds to the transition state. [Color figure can be viewed at wileyonlinelibrary.com]

Figure 4 shows profiles of the *total* intra‐atomic and interatomic energies in the four reactions.

The profiles of the variation of the total intra‐atomic and interatomic energies are similar for all four reactions. At the first stages of the reaction, a stabilization of the total interatomic energy (dotted lines) and destabilization of the intra‐atomic energies (dashed lines) is observed. This trend changes as the reaction advances, and the two curves cross at ξ = −2.2, −2.2, −2.4, and −3.1 for X = F, Cl, Br, and I, respectively. After this point, and continuing to move to the right, the values of the interatomic energies are destabilizing while the intra‐atomic ones are stabilizing. In the entire reaction coordinate the sum of these two energy terms provides a net positive balance, in agreement with the reaction barrier observed. Hence, overall the reaction barriers are due to interatomic energies destabilizing the transition states more than the intra‐atomic energies can stabilize them.

#### Atomic contributions to *E*_intra_ and *V*_inter_

**Figure 5:**
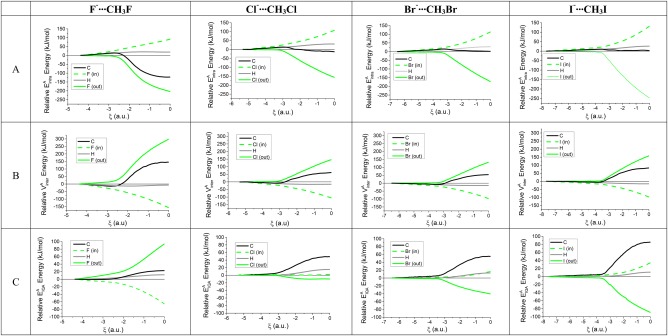
Variation in atomic resolution of a) the intra‐atomic energies $E_{\text{intra}}^{A}$, b) the interatomic (potential) energies $V_{\text{inter}}^{A}$, and c) the total atomic energies $E_{\text{IQA}}^{A}$for all four reactions and all atoms. The energetic profile of H corresponds to the sum of the contributions of the three degenerate H atoms. The color legend is natural in that Carbon is black, the Halogen green and the Hydrogen gray (for clear contrast, instead of the conventional white). [Color figure can be viewed at wileyonlinelibrary.com]

Figure 5 plots the *atomic* contributions to the total energy( $E_{\text{IQA}}^{A}$), intra‐atomic ( $E_{\text{intra}}^{A}$) and interatomic energies ( $V_{\text{inter}}^{A}$) along the reaction coordinate. Table 1 accompanies Figure 5, listing the differences in the respective energies of the energy minimum and the TS. The overall shape of the $E_{\text{intra}}^{A}$, $E_{\text{IQA}}^{A}$, and $V_{\text{inter}}^{A}$ profiles for any atom *A* in any of the four reactions is very similar. However, the F profiles again distinguish themselves from the profiles of Cl, Br, and I. Starting with the intra‐atomic energies (Panel A in Fig. 5), the variation (going from minimum to TS) of $E_{\text{intra}}^{C}$ (black) in F^–^···CH_3_F is highly stabilizing (–122 kJ/mol) while in the other reactions it is close to zero. Next, we look at $V_{\text{inter}}^{A}$(Panel B) and find that the variation observed for all the nonhydrogen atoms in F^–^···CH_3_F is much larger in absolute value than that found in the remaining reactions. Finally, Panel C ( $E_{\text{IQA}}^{A}$) reveals that the variation of the total atomic energies in F^–^···CH_3_F is stabilizing for *F*_in_ and destabilizing for *F*_out_. In contrast, in the rest of the reactions, the behavior is just the opposite: *X*_in_ is slightly destabilizing and *X*_out_ is stabilizing. Another main difference in the total atomic energies is the contribution of the carbon atom, which is small in the F^–^···CH_3_F case but the most import destabilizing contribution in the rest of the reactions.

**Table 1:** Differences of the atomic total (Δ*E*_IQA_), intra‐atomic (Δ*E*_intra_), and interatomic energies (Δ*V*_inter_; kJ/mol) between the energy minimum and the transition state.

	F^–^···CH_3_F			Cl^–^···CH_3_Cl			Br^–^···CH_3_Br			I^–^···CH_3_I		
	Δ*E*_IQA_	Δ*E*_intra_	Δ*V*_inter_	Δ*E*_IQA_	Δ*E*_intra_	Δ*V*_inter_	Δ*E*_IQA_	Δ*E*_intra_	Δ*V*_inter_	Δ*E*_IQA_	Δ*E*_intra_	Δ*V*_inter_
**C**	23	−122	145	49	−14	63	55	−1	56	85	0	85
***X*_**in**_**	−66	91	−157	2	108	−106	16	114	−97	34	132	−98
**H** a	12	19	−7	15	31	−15	13	28	−15	11	26	−15
***X*_**out**_**	94	−204	298	−10	−156	146	−40	−172	132	−89	−248	159
**All**	63	−216	278	56	−32	88	44	−32	76	41	−90	131

a Variation of the three H atoms together, that is, the hydrogen's energies have been summed.

In the Cl, Br, I series the $E_{\text{intra}}^{X}$ and $E_{\text{IQA}}^{X}$ values are stabilized for the *X*_in_ and destabilized for the *X*_out_, increasing their absolute values with increasing halogen atom size.

The evolution of the systems from the energy minima to the TSs (ξ = 0) produces important changes in the electronic structure. A simple exploration of the atomic charges (Table 2) shows that while *X*_in_ in the minimum presents charges close to −1 e, they are reduced in magnitude by 0.19, 0.25, 0.27, and 0.30 e (for X= F, Cl, Br, and I, respectively) when the TS is reached. In the F^–^···CH_3_F system, the charge gain is divided between the C and *F*_out_ atoms while, in the rest of the systems, all charge mostly migrates to the *X*_out_ atom. These changes should affect the values of *E*_intra_ for each atom as well as *V*_inter_. The results obtained here are similar to those reported by Joubert et al. in the Cl^–^···CH_3_Cl reaction studied at PBE0/6–31+G(d,p) level. They report a charge transfer of 0.25 e for the Cl_in_ in going from the minimum to the TS, and a charge of −0.70 e for the Cl atoms in the TS.^43^

**Table 2:** Atomic charges (e) at the minimum energy and TS, and the difference between the TS and the minimum.

	F^–^···CH_3_F			Cl^–^···CH_3_Cl			Br^–^···CH_3_Br			I^–^···CH_3_I		
	Min	TS	Δ	Min	TS	Δ	Min	TS	Δ	Min	TS	Δ
**C**	0.492	0.406	−0.086	0.102	0.115	0.013	−0.004	0.056	0.060	−0.144	−0.023	0.121
**H** a	0.152	0.139	−0.013	0.226	0.273	0.047	0.240	0.283	0.043	0.235	0.281	0.045
***X*_**out**_**	−0.681	−0.772	−0.091	−0.381	−0.694	−0.313	−0.298	−0.670	−0.372	−0.161	−0.629	−0.468
***X*_**in**_**	−0.963	−0.772	0.191	−0.947	−0.694	0.253	−0.938	−0.670	0.268	−0.930	−0.629	0.301

a Variation of the three H atoms summed.

#### Interatomic terms

Although the systems considered are very simple, seven unique intermolecular energy terms are needed to describe all the atom–atom energy contributions. Each of these contributions has been divided in classical (*V*_cl_) and nonclassical (*V*_xc_, exchange‐correlation) according to eq. (5). Table 3 lists the all these energy contributions for the seven unique intermolecular energy contributions.

**Table 3:** Variation of the interatomic terms (kJ/mol) between the energy minimum and the TS.

	F^–^···CH_3_F			Cl^–^···CH_3_Cl			Br^–^···CH_3_Br			I^–^···CH_3_I		
	Δ*V*_inter_	Δ*V*_cl_	Δ*V*_xc_	Δ*V*_inter_	Δ*V*_cl_	Δ*V*_xc_	Δ*V*_inter_	Δ*V*_cl_	Δ*V*_xc_	Δ*V*_inter_	Δ*V*_cl_	Δ*V*_xc_
**C**—***X*_**out**_**	596	321	274	325	−60	386	302	−60	362	360	7	353
**C**—***X*_**in**_**	−278	−20	−258	−226	−5	−221	−228	−17	−211	−244	−36	−208
***X*_**out**_**—***X*_**in**_**	−25	−16	−9	27	39	−12	40	52	−13	56	70	−14
**C**—**H** a	−9	−1	−8	7	16	−9	12	19	−8	17	24	−8
***X*_**out**_**—**H** a	7	7	0	−20	−20	1	−26	−26	0	−34	−33	−1
**H**—***X*_**in**_** a	−4	2	−6	−4	0	−5	−1	3	−4	3	6	−3
**H**—**H’** a	0	−2	2	3	2	1	2	1	1	3	2	1

a Three identical terms are obtained for each system.

The variation of the carbon–halogen interatomic energy terms is by far the most important one for each reactive system. In all four cases, a destabilization is observed in the C—*X*_out_ terms whilst progressing from the energy minimum to the TS, as expected due to the elongation (weakening) of such bonds. In contrast, the C—*X*_in_ terms are stabilized, as expected for the formation of such bonds. In all cases, Δ*V*_xc_ is more important than Δ*V*_cl_ except in the C—*F*_out_ term, where the opposite happens. The overall balance of these C—*X*_in_ and C—*X*_out_ interatomic terms is destabilizing.

The third most important interatomic term corresponds to the interaction between the two halogen atoms. The Δ*V*_xc_ component is in all cases negative while Δ*V*_cl_ is positive for X = Cl, Br and I but negative for X = F. The value of Δ*V*_inter_ is negative for the F^–^···CH_3_F reaction (–25 kJ/mol) while positive for the rest of the systems, increasing its value with the size of the halogen atom (27, 40, and 56 for X = Cl, Br, and I).

#### Analysis by the REG method

We used the REG method to analyze the variation of the intra‐atomic and interatomic (classical and exchange‐correlations) energy terms in the four reactions *versus* the total energy profile. Because there are 6 atoms in each system there are 6 intra‐atomic and twice 6×(6‐1)/2 = 15 interatomic energy terms, one set for *V*_cl_ and one set for *V*_xc_, totaling 36 = 6 + 15 + 15 energy terms. However, because of the degeneracy of the three hydrogen atoms, there are fewer unique energy terms than the number of 36. In fact, there are only 18 = 4 + 7 + 7 unique terms, where the seven unique interatomic terms are listed in Table 3. However, we chose to report on all 36 terms, thereby verifying that the ANANKE program works correctly when returning identical values for degenerate atoms.

Table 4 gathers the energetic terms with the three highest and the three lowest (in real, not absolute, terms) REG values. The complete table is available in the Supporting Information (Table S3). The three positive terms listed in Table 4 represent between 80% and 93% of the sum of all the positive terms in each reaction, and for the negative ones, between 73% and 88%. In addition, all terms listed show good linear relationships with the variation of the total energy (|*R*| > 0.97). Note that the |*R*| values drop considerably for the energy term ranked 4th and 33rd, to below 0.8 in absolute value, and most dramatically so for F. This sudden drop strongly supports the fact that the total energy reaction profiles can be understood from only six energy terms, three of which work in the same direction and with the energy barrier, and the other three in the opposing direction.

**Table 4:** The relative energy gradient (REG) and Pearson coefficient (*R*) of the largest three positive and the largest three negative terms, as calculated by the program ANANKE.

	F···CH_3_F			Cl···CH_3_Cl			Br···CH_3_Br			I···CH_3_I		
Rank	REG	R	Term	REG	R	Term	REG	R	Term	REG	R	Term
**1**	5.6	0.99	*V*_cl_ C—*F*_out_	6.3	1.00	*V*_xc_ C—Cl_out_	7.8	0.99	*V*_xc_ C—Br_out_	7.7	0.99	*V*_xc_ C—I_out_
**2**	3.7	0.98	*V*_xc_ C—*F*_out_	1.5	0.99	*E*_intra_ Cl_in_	2.1	0.99	*E*_intra_ Br_in_	2.4	0.99	*E*_intra_ I_in_
**3**	1.2	0.98	*E*_intra_ *F*_in_	0.7	1.00	*V*_cl_ Cl_in_—Cl_out_	1.3	0.99	*V*_cl_ Br_in_—Br_out_	1.8	0.99	*V*_cl_ I_in_—I_out_
**34**	−2.4	−0.98	*E*_intra_ C	−1.1	−0.99	*V*_cl_ C—Cl_out_	−1.5	−0.99	*V*_cl_ C—Br_out_	−1.0	−0.97	*V*_cl_ C—I_in_
**35**	−3.3	−0.98	*V*_xc_ C—*F*_in_	−2.6	−1.00	*E*_intra_ Cl_out_	−3.7	−0.98	*V*_xc_ C—Br_in_	−3.7	−0.98	*V*_xc_ C—I_in_
**36**	−3.4	−0.99	*E*_intra_ *F*_out_	−3.0	−0.97	*V*_xc_ C—Cl_in_	−3.8	−1.00	*E*_intra_ Br_out_	−5.7	−0.99	*E*_intra_ I_out_

The REG values of the highest ranked energy terms are much higher than the values of the ones ranked second. This is why the highest ranked terms are a good starting point for the discussion of Table 4. A first important insight is that all halogens except F return the *V*_xc_(C—*X*_out_) energy term as the strongest contributor to the transition energy barrier. As the system progresses toward the transition state, the C—*X*_out_ bond length increases and the bond weakens. This destabilization is the main contributor toward the transition energy barrier for all systems except the F^–^···CH_3_F system. To be more precise, this effect does exist in F^–^···CH_3_F but it is not as strong a trend, which is why it has only the second highest REG value. However, the energy term that does dominate F^–^···CH_3_F is *V*_cl_(C—*F*_out_). This finding also makes sense because the atomic charges of C and *F*_out_ are large, and change little along the reaction coordinate (Table 2), leading to a strong electrostatic interaction that decreases as the C—*X*_out_ bond length again increases.

A second important chemical insight to be gained from Table 4 is coming from the role of *E*_intra_(*X*_in_), which is always ranked second in terms of REG value, again for all halogens except F, where it is third. A recent paper^88^ from our group has shown conclusively that *E*_intra_ is linked to intermolecular repulsion, that is, the steric effect that is captured by classical repulsive potentials such as the Buckingham potential. In the current context of S_N_2 reactions, the incoming ion must be interpreted as helping the construction of the reaction barrier, by its steric repulsion, as it approaches the carbon in the transition state. Using a metaphor, the work in paper^88^ showed that (topological atoms) act more like sponges rather than billiard balls. In other words, on approach (from infinity) two topological atoms interact “sooner” than hard‐sphere‐type atoms. Note that the latter come with empty spaces (as in a ball‐and‐stick picture), while the former are space‐filling (i.e., no empty space). In summary, we use the qualifier spongy to refer to atoms that deform at large distances away from other atoms. This view is confirmed by the fact that their *E*_intra_ values fit the Buckingham potential better than the Lennard–Jones potential. Indeed, the latter supports a billiard ball picture while the former support a sponge. Within this view, we can then state one more assertion: F acts like a hard sponge displaying smaller deformation energies. In other words, the harder F resists deformation while the softer I prefers to absorb a lot of deformation. This finding explains the fact that *E*_intra_(*F*_in_) is ranked third rather than second. Table 1 shows that the *E*_intra_(*X*_in_) values monotonically increase going from F to I, confirming a gradient of steric interaction, which adopts its highest value for I. This observation in turns relates to I being the most deformable sponge, causing the largest deformation energy in the transition state.

The intra‐atomic energies can be further analysed in two more ways (the raw data for which are in Supporting Information Table S4): (i) the role of kinetic energy (which is part of the intra‐atomic energy, alongside Coulomb and exchange energy), and (ii) a segmented analysis (of a single barrier) where there is a “far segment” (i.e., furthest from the transition state, with ξ < −3, approximately, depending on the halogen) and “close segment” (i.e., nearest to the transition state, with ξ > −3, approximately). Pleasingly, this new refined analysis returns a clear picture for both segments: the kinetic energy dominates the onset of the barrier in the far segment, while in the close segment, the exchange‐correlation dominates, except for F where the electrostatics dominate.

The third and final insight is more subtle and derives from the *V*_cl_(*X*_in_—*X*_out_) energy term. Hence, the electrostatic interaction between incoming and outgoing halogen (again except for F) works in the same direction as the energy barrier. To understand this finding in simpler terms one can bring in atomic charges from Table 2 and approximate *V*_cl_ values. The sum of the charges (Table 2) in the halogen atoms increases in absolute value in the transition state for Cl, Br, and I. Thus, the variation in *V*_cl_(*X*_in_—*X*_out_) is positive for Cl, Br, and I, thereby helping the barrier. This explanation works for F but in the opposite way, and only weakly, which is why it is ranked 33rd (see Supporting Information Table S3).

Similar arguments can be applied to retrieve chemical insight from the most negative REG values (in Table 4). For example, *E*_intra_(*X*_out_) indicates that the egression of *X*_out_ reduces steric repulsion (as the reaction proceeds toward the transition) and hence works against the energy barrier. Second, *V*_xc_(C—*X*_in_) is easy to understand because the formation of a bond between the incoming halogen and the carbon also works against the energy barrier.

Finally, none of the important terms involves the hydrogen atoms for positive and negative REG values.

## Conclusions

A computational study of the prototypical S_N_2 reaction X^–^ + CH_3_X → XCH_3_ + X^–^ with X= F, Cl, Br, and I has been carried out using QTAIM and IQA, which together are part of the QCT *Ansatz*. In all four reactions, the energy barrier at the transition state is due to an interatomic destabilization that fails to be compensated by an opposing intra‐atomic stabilization. However, in the early stage of any of the four reactions, the growing energy barrier is due to dominance of the intra‐atomic energy.

Although the four reactions share many common characteristics, the reaction involving F has a number of particularities that differentiate it from the rest.

The REG method (implemented in the *ANANKE* program) enables to rank IQA energy contributions according to their importance in constructing a reaction barrier, or working against it. As such it is able to extract chemical insight from a reaction, both in terms of atomic locale and energy type (steric, exchange, and electrostatic). The REG method shows that four (*V*_xc_(C—*X*_in_), *V*_xc_(C—*X*_out_), *E*_intra_(*X*_in_), and *E*_intra_(*X*_out_)) of the six most important energetic terms are *common to all* the reactions in explaining their reaction barriers.

## Supporting information

Supporting Information
