# Supplementary material for: An interacting quantum atom study of model SN2 reactions (X–···CH3X, X = F, Cl, Br, and I)
Source: J Comput Chem. 2017 Nov 10;39(10):546–56. doi: 10.1002/jcc.25098 (PMC5836863; doi:10.1002/jcc.25098)
Supplement: Supplementary file 1 — Supporting Information [file JCC-39-546-s001.doc]

**Supporting Information**

**An Interacting Quantum Atom (IQA) study of model S_N_2 reactions (X^‑^···CH_3_X, X=F, Cl, Br and I)**

Ibon Alkorta* ^a^, Joseph C.R. Thacker^b^ and Paul L.A. Popelier* ^b^

^a^ Instituto de Química Médica (CSIC), Juan de la Cierva, 3, 28006-Madrid, Spain

^b^ Manchester Institute of Biotechnology (MIB), 131 Princess Street, M1 7DN, Great Britain, and School of Chemistry, University of Manchester, Oxford Road, Manchester M13 9PL, Great Britain

**Content**

Pg. S2-3 **Table S1.**

Electronic energy, number of imaginary frequencies and optimized of the stationary points.

Pg. S4-12 **Table S2.**

Electron density properties (au) of the C-X BCPs along the reaction coordinate.

Pg. S13-14 **Table S3**.

The Relative Energy Gradient (REG) and Pearson coefficient (R) as calculated by

the program ANANKE.

Pg. S15-18 **Table S4 + Discussion**.

The Relative Energy Gradient (REG) and Pearson coefficient (R) as calculated by the

program ANANKE. Refined analysis (i.e. less coarse-grained than Table S3): (i) breakdown of

Intra-atomic energies (Coulomb, exchange and kinetic energy (KE)); (ii) division of reaction

barrier into two segments: close segment (ξ > -2.9) and far segment (ξ < -2.9)

(but for I ξ = -3.3 is the segment boundary).

**Table S1.** Electronic energy, number of imaginary frequencies and optimized geometries of the stationary points.

| FCH_3_F (min)  Electronic Energy= -239.598205617 Hartree, NIMAG= 0  C  F,1,r0  H,1,r1,2,a1  H,1,r1,2,a1,3,120.,0  H,1,r1,2,a1,3,-120.,0  X,1,1.,2,90.,3,0.,0  F,1,r2,6,90.,3,0.,0  r0=1.4289309  r1=1.08417189  a1=108.86262226  r2=2.52881057 | FCH_3_F (TS)  Electronic Energy= -239.574288829 Hartree, NIMAG= 1  C  F,1,r0  H,1,r1,2,90.  H,1,r1,2,90.,3,120.,0  H,1,r1,2,90.,3,-120.,0  F,1,r0,3,90.,2,180.,0  r0=1.80392805  r1=1.07489799 |
| --- | --- |
| ClCH3Cl (min)  Electronic Energy= -960.379655269 Hartree, NIMAG= 0  C  Cl,1,r0  H,1,r1,2,a1  H,1,r1,2,a1,3,120.,0  H,1,r1,2,a1,3,-120.,0  X,1,1.,2,90.,3,0.,0  Cl,1,r2,6,90.,3,0.,0  r0=1.82807876  r1=1.08278496  a1=108.39927839  r2=3.11747094 | ClCH3Cl (TS)  Electronic Energy= -960.358158620 Hartree, NIMAG= 1  C  Cl,1,r0  H,1,r1,2,90.  H,1,r1,2,90.,3,120.,0  H,1,r1,2,90.,3,-120.,0  Cl,1,r0,3,90.,2,180.,0  r0=2.31298368  r1=1.07293536 |
| BrCH3Br (min)  Electronic Energy= -5188.34792686 Hartree, NIMAG= 0  C  Br,1,r0  H,1,r1,2,a1  H,1,r1,2,a1,3,120.,0  H,1,r1,2,a1,3,-120.,0  X,1,1.,2,90.,3,0.,0  Br,1,r2,6,90.,3,0.,0  r0=1.98629804  r1=1.08220105  a1=107.6015667  r2=3.25344312 | BrCH3Br (TS)  Electronic Energy= -5188.33127324 Hartree, NIMAG= 1  C  Br,1,r0  H,1,r1,2,90.  H,1,r1,2,90.,3,120.,0  H,1,r1,2,90.,3,-120.,0  Br,1,r0,3,90.,2,180.,0  r0=2.4624388  r1=1.07339821 |

| ICH3I (min)  Electronic Energy= -13879.8855695 Hartree, NIMAG= 0  C  I,1,r0  H,1,r1,2,a1  H,1,r1,2,a1,3,120.,0  H,1,r1,2,a1,3,-120.,0  X,1,1.,2,90.,3,0.,0  I,1,r2,6,90.,3,0.,0  r0=2.17276038  r1=1.08267096  a1=107.61618216  r2=3.48595741 | ICH3I (TS)  Electronic Energy= -13879.8691894 Hartree, NIMAG= 1  C  I,1,r0  H,1,r1,2,90.  H,1,r1,2,90.,3,120.,0  H,1,r1,2,90.,3,-120.,0  I,1,r0,3,90.,2,180.,0  r0=2.65998991  r1=1.07395797 |
| --- | --- |

**Table S2.** Electron density properties (au) of the C-X BCPs along the reaction coordinate.

| **FCH_3_F** | C-F_out_ | | | | C-F_in_ | | | |
| --- | --- | --- | --- | --- | --- | --- | --- | --- |
| IRC | C-F dist. (Å) | ρ_BCP_ | ∇^2^ρ_BCP_ | H_BCP_ | C-F dist. (Å) | ρ_BCP_ | ∇^2^ρ_BCP_ | H_BCP_ |
| -4.37 | 1.430 | 0.2020 | 0.1558 | -0.2460 | 2.517 | 0.0193 | 0.0876 | 0.0015 |
| -4.30 | 1.429 | 0.2021 | 0.1576 | -0.2460 | 2.507 | 0.0197 | 0.0895 | 0.0015 |
| -4.22 | 1.430 | 0.2019 | 0.1566 | -0.2457 | 2.497 | 0.0201 | 0.0915 | 0.0015 |
| -4.15 | 1.430 | 0.2019 | 0.1583 | -0.2457 | 2.486 | 0.0205 | 0.0936 | 0.0015 |
| -4.08 | 1.430 | 0.2017 | 0.1574 | -0.2454 | 2.475 | 0.0209 | 0.0957 | 0.0015 |
| -4.01 | 1.430 | 0.2016 | 0.1575 | -0.2452 | 2.465 | 0.0214 | 0.0978 | 0.0016 |
| -3.93 | 1.430 | 0.2015 | 0.1572 | -0.2449 | 2.454 | 0.0218 | 0.1001 | 0.0016 |
| -3.86 | 1.431 | 0.2013 | 0.1571 | -0.2447 | 2.443 | 0.0223 | 0.1023 | 0.0016 |
| -3.79 | 1.431 | 0.2012 | 0.1568 | -0.2445 | 2.433 | 0.0227 | 0.1047 | 0.0016 |
| -3.71 | 1.431 | 0.2011 | 0.1566 | -0.2442 | 2.422 | 0.0232 | 0.1071 | 0.0016 |
| -3.64 | 1.431 | 0.2009 | 0.1563 | -0.2440 | 2.411 | 0.0237 | 0.1095 | 0.0016 |
| -3.57 | 1.431 | 0.2008 | 0.1560 | -0.2437 | 2.401 | 0.0242 | 0.1120 | 0.0016 |
| -3.50 | 1.432 | 0.2007 | 0.1557 | -0.2435 | 2.390 | 0.0247 | 0.1146 | 0.0017 |
| -3.42 | 1.432 | 0.2005 | 0.1553 | -0.2432 | 2.379 | 0.0252 | 0.1173 | 0.0017 |
| -3.35 | 1.432 | 0.2004 | 0.1549 | -0.2430 | 2.368 | 0.0257 | 0.1200 | 0.0017 |
| -3.28 | 1.432 | 0.2002 | 0.1545 | -0.2427 | 2.358 | 0.0263 | 0.1228 | 0.0017 |
| -3.20 | 1.433 | 0.2001 | 0.1541 | -0.2425 | 2.347 | 0.0268 | 0.1256 | 0.0017 |
| -3.13 | 1.433 | 0.1999 | 0.1535 | -0.2422 | 2.336 | 0.0274 | 0.1285 | 0.0017 |
| -3.06 | 1.433 | 0.1998 | 0.1526 | -0.2419 | 2.325 | 0.0280 | 0.1315 | 0.0018 |
| -2.99 | 1.434 | 0.1996 | 0.1515 | -0.2415 | 2.315 | 0.0286 | 0.1346 | 0.0018 |
| -2.91 | 1.434 | 0.1993 | 0.1498 | -0.2411 | 2.304 | 0.0292 | 0.1378 | 0.0018 |
| -2.84 | 1.435 | 0.1990 | 0.1473 | -0.2405 | 2.293 | 0.0298 | 0.1410 | 0.0018 |
| -2.77 | 1.436 | 0.1986 | 0.1437 | -0.2398 | 2.282 | 0.0305 | 0.1444 | 0.0018 |
| -2.70 | 1.437 | 0.1980 | 0.1386 | -0.2388 | 2.270 | 0.0312 | 0.1478 | 0.0018 |
| -2.62 | 1.439 | 0.1973 | 0.1316 | -0.2375 | 2.259 | 0.0319 | 0.1514 | 0.0018 |
| -2.55 | 1.441 | 0.1963 | 0.1220 | -0.2358 | 2.247 | 0.0327 | 0.1550 | 0.0018 |
| -2.48 | 1.444 | 0.1950 | 0.1094 | -0.2335 | 2.235 | 0.0336 | 0.1587 | 0.0018 |
| -2.40 | 1.449 | 0.1934 | 0.0933 | -0.2305 | 2.222 | 0.0344 | 0.1625 | 0.0018 |
| -2.33 | 1.454 | 0.1914 | 0.0739 | -0.2267 | 2.209 | 0.0354 | 0.1663 | 0.0018 |
| -2.26 | 1.460 | 0.1890 | 0.0517 | -0.2220 | 2.196 | 0.0363 | 0.1700 | 0.0017 |
| -2.19 | 1.467 | 0.1864 | 0.0280 | -0.2164 | 2.182 | 0.0373 | 0.1736 | 0.0017 |
| -2.11 | 1.475 | 0.1834 | 0.0042 | -0.2098 | 2.169 | 0.0384 | 0.1772 | 0.0016 |
| -2.04 | 1.484 | 0.1803 | -0.0178 | -0.2023 | 2.156 | 0.0395 | 0.1806 | 0.0015 |
| -1.97 | 1.493 | 0.1770 | -0.0361 | -0.1940 | 2.142 | 0.0406 | 0.1838 | 0.0014 |
| -1.89 | 1.503 | 0.1735 | -0.0495 | -0.1848 | 2.129 | 0.0417 | 0.1869 | 0.0012 |
| -1.82 | 1.514 | 0.1700 | -0.0571 | -0.1751 | 2.116 | 0.0429 | 0.1898 | 0.0010 |
| -1.75 | 1.524 | 0.1665 | -0.0591 | -0.1651 | 2.103 | 0.0442 | 0.1926 | 0.0008 |
| -1.68 | 1.535 | 0.1629 | -0.0560 | -0.1550 | 2.090 | 0.0455 | 0.1951 | 0.0006 |
| -1.60 | 1.546 | 0.1593 | -0.0489 | -0.1451 | 2.077 | 0.0468 | 0.1974 | 0.0003 |
| -1.53 | 1.557 | 0.1557 | -0.0388 | -0.1355 | 2.064 | 0.0482 | 0.1994 | -0.0001 |
| -1.46 | 1.568 | 0.1521 | -0.0266 | -0.1262 | 2.051 | 0.0497 | 0.2012 | -0.0005 |
| -1.38 | 1.580 | 0.1485 | -0.0131 | -0.1175 | 2.038 | 0.0513 | 0.2027 | -0.0009 |
| -1.31 | 1.591 | 0.1449 | 0.0010 | -0.1092 | 2.026 | 0.0529 | 0.2040 | -0.0014 |
| -1.24 | 1.603 | 0.1414 | 0.0154 | -0.1014 | 2.013 | 0.0545 | 0.2049 | -0.0020 |
| -1.17 | 1.614 | 0.1379 | 0.0297 | -0.0941 | 2.001 | 0.0563 | 0.2055 | -0.0027 |
| -1.09 | 1.626 | 0.1345 | 0.0437 | -0.0872 | 1.988 | 0.0581 | 0.2058 | -0.0034 |
| -1.02 | 1.637 | 0.1311 | 0.0572 | -0.0808 | 1.976 | 0.0600 | 0.2058 | -0.0042 |
| -0.95 | 1.649 | 0.1277 | 0.0702 | -0.0748 | 1.963 | 0.0619 | 0.2055 | -0.0051 |
| -0.87 | 1.661 | 0.1245 | 0.0825 | -0.0693 | 1.951 | 0.0639 | 0.2048 | -0.0060 |
| -0.80 | 1.673 | 0.1212 | 0.0941 | -0.0640 | 1.938 | 0.0660 | 0.2039 | -0.0071 |
| -0.73 | 1.684 | 0.1181 | 0.1050 | -0.0592 | 1.926 | 0.0681 | 0.2026 | -0.0082 |
| -0.66 | 1.696 | 0.1150 | 0.1152 | -0.0546 | 1.914 | 0.0703 | 0.2011 | -0.0095 |
| -0.58 | 1.708 | 0.1119 | 0.1247 | -0.0504 | 1.902 | 0.0726 | 0.1993 | -0.0108 |
| -0.51 | 1.720 | 0.1089 | 0.1334 | -0.0465 | 1.889 | 0.0748 | 0.1971 | -0.0122 |
| -0.44 | 1.732 | 0.1060 | 0.1415 | -0.0428 | 1.877 | 0.0772 | 0.1947 | -0.0137 |
| -0.36 | 1.744 | 0.1031 | 0.1489 | -0.0394 | 1.865 | 0.0796 | 0.1920 | -0.0154 |
| -0.29 | 1.756 | 0.1003 | 0.1557 | -0.0362 | 1.853 | 0.0820 | 0.1889 | -0.0171 |
| -0.22 | 1.768 | 0.0975 | 0.1619 | -0.0332 | 1.840 | 0.0845 | 0.1855 | -0.0190 |
| -0.15 | 1.780 | 0.0948 | 0.1676 | -0.0304 | 1.828 | 0.0870 | 0.1816 | -0.0210 |
| -0.07 | 1.792 | 0.0922 | 0.1727 | -0.0278 | 1.816 | 0.0895 | 0.1774 | -0.0231 |
| 0.00 | 1.804 | 0.0895 | 0.1774 | -0.0254 | 1.804 | 0.0189 | 0.0857 | -0.0254 |

| **ClCH_3_Cl** | C-Cl_out_ | | | | C-Cl_in_ | | | |
| --- | --- | --- | --- | --- | --- | --- | --- | --- |
| IRC | C-Cl dist. (Å) | ρ_BCP_ | ∇^2^ρ_BCP_ | H_BCP_ | C-Cl dist. (Å) | ρ_BCP_ | ∇^2^ρ_BCP_ | H_BCP_ |
| -5.32 | 1.829 | 0.1608 | -0.1811 | -0.1027 | 3.089 | 0.0116 | 0.0441 | 0.0018 |
| -5.24 | 1.828 | 0.1611 | -0.1820 | -0.1031 | 3.081 | 0.0117 | 0.0448 | 0.0018 |
| -5.17 | 1.828 | 0.1610 | -0.1816 | -0.1031 | 3.072 | 0.0119 | 0.0455 | 0.0018 |
| -5.10 | 1.828 | 0.1610 | -0.1816 | -0.1031 | 3.064 | 0.0121 | 0.0463 | 0.0018 |
| -5.03 | 1.829 | 0.1608 | -0.1808 | -0.1028 | 3.055 | 0.0123 | 0.0471 | 0.0018 |
| -4.95 | 1.828 | 0.1609 | -0.1811 | -0.1030 | 3.047 | 0.0125 | 0.0479 | 0.0019 |
| -4.88 | 1.828 | 0.1607 | -0.1806 | -0.1029 | 3.038 | 0.0127 | 0.0487 | 0.0019 |
| -4.81 | 1.828 | 0.1608 | -0.1808 | -0.1030 | 3.030 | 0.0128 | 0.0495 | 0.0019 |
| -4.73 | 1.828 | 0.1607 | -0.1804 | -0.1029 | 3.021 | 0.0130 | 0.0503 | 0.0019 |
| -4.66 | 1.828 | 0.1608 | -0.1806 | -0.1030 | 3.013 | 0.0132 | 0.0511 | 0.0019 |
| -4.59 | 1.828 | 0.1607 | -0.1801 | -0.1029 | 3.004 | 0.0134 | 0.0520 | 0.0019 |
| -4.52 | 1.828 | 0.1608 | -0.1803 | -0.1031 | 2.996 | 0.0136 | 0.0528 | 0.0019 |
| -4.44 | 1.828 | 0.1609 | -0.1805 | -0.1033 | 2.988 | 0.0138 | 0.0537 | 0.0019 |
| -4.37 | 1.828 | 0.1608 | -0.1801 | -0.1031 | 2.979 | 0.0140 | 0.0546 | 0.0019 |
| -4.30 | 1.828 | 0.1607 | -0.1799 | -0.1031 | 2.971 | 0.0143 | 0.0555 | 0.0019 |
| -4.22 | 1.828 | 0.1607 | -0.1798 | -0.1032 | 2.962 | 0.0145 | 0.0564 | 0.0019 |
| -4.15 | 1.828 | 0.1607 | -0.1798 | -0.1032 | 2.954 | 0.0147 | 0.0573 | 0.0019 |
| -4.08 | 1.828 | 0.1608 | -0.1797 | -0.1033 | 2.945 | 0.0149 | 0.0583 | 0.0019 |
| -4.01 | 1.827 | 0.1608 | -0.1796 | -0.1033 | 2.937 | 0.0151 | 0.0592 | 0.0020 |
| -3.93 | 1.827 | 0.1608 | -0.1797 | -0.1034 | 2.928 | 0.0154 | 0.0602 | 0.0020 |
| -3.86 | 1.827 | 0.1608 | -0.1795 | -0.1034 | 2.920 | 0.0156 | 0.0611 | 0.0020 |
| -3.79 | 1.827 | 0.1609 | -0.1796 | -0.1035 | 2.912 | 0.0158 | 0.0621 | 0.0020 |
| -3.71 | 1.827 | 0.1608 | -0.1792 | -0.1035 | 2.903 | 0.0161 | 0.0631 | 0.0020 |
| -3.64 | 1.827 | 0.1609 | -0.1796 | -0.1037 | 2.895 | 0.0163 | 0.0641 | 0.0020 |
| -3.57 | 1.827 | 0.1609 | -0.1792 | -0.1037 | 2.886 | 0.0166 | 0.0651 | 0.0020 |
| -3.50 | 1.827 | 0.1609 | -0.1793 | -0.1037 | 2.878 | 0.0168 | 0.0662 | 0.0020 |
| -3.42 | 1.827 | 0.1607 | -0.1782 | -0.1035 | 2.869 | 0.0171 | 0.0672 | 0.0019 |
| -3.35 | 1.827 | 0.1605 | -0.1776 | -0.1033 | 2.860 | 0.0173 | 0.0684 | 0.0019 |
| -3.28 | 1.828 | 0.1602 | -0.1762 | -0.1029 | 2.851 | 0.0176 | 0.0695 | 0.0019 |
| -3.20 | 1.830 | 0.1596 | -0.1742 | -0.1022 | 2.841 | 0.0179 | 0.0707 | 0.0019 |
| -3.13 | 1.833 | 0.1588 | -0.1710 | -0.1011 | 2.831 | 0.0183 | 0.0719 | 0.0019 |
| -3.06 | 1.836 | 0.1575 | -0.1664 | -0.0995 | 2.820 | 0.0186 | 0.0732 | 0.0019 |
| -2.99 | 1.842 | 0.1557 | -0.1602 | -0.0972 | 2.809 | 0.0190 | 0.0746 | 0.0019 |
| -2.91 | 1.848 | 0.1535 | -0.1525 | -0.0945 | 2.797 | 0.0194 | 0.0759 | 0.0018 |
| -2.84 | 1.856 | 0.1509 | -0.1437 | -0.0912 | 2.784 | 0.0199 | 0.0773 | 0.0018 |
| -2.77 | 1.866 | 0.1481 | -0.1341 | -0.0878 | 2.772 | 0.0203 | 0.0786 | 0.0018 |
| -2.70 | 1.875 | 0.1451 | -0.1242 | -0.0841 | 2.759 | 0.0208 | 0.0800 | 0.0017 |
| -2.62 | 1.886 | 0.1419 | -0.1142 | -0.0805 | 2.747 | 0.0213 | 0.0812 | 0.0017 |
| -2.55 | 1.896 | 0.1387 | -0.1042 | -0.0768 | 2.734 | 0.0218 | 0.0824 | 0.0016 |
| -2.48 | 1.908 | 0.1356 | -0.0944 | -0.0732 | 2.722 | 0.0223 | 0.0836 | 0.0015 |
| -2.40 | 1.919 | 0.1324 | -0.0848 | -0.0697 | 2.710 | 0.0228 | 0.0847 | 0.0015 |
| -2.33 | 1.930 | 0.1292 | -0.0754 | -0.0663 | 2.698 | 0.0234 | 0.0858 | 0.0014 |
| -2.26 | 1.942 | 0.1260 | -0.0664 | -0.0630 | 2.685 | 0.0239 | 0.0868 | 0.0013 |
| -2.19 | 1.953 | 0.1229 | -0.0577 | -0.0599 | 2.673 | 0.0245 | 0.0878 | 0.0012 |
| -2.11 | 1.965 | 0.1199 | -0.0493 | -0.0568 | 2.661 | 0.0251 | 0.0887 | 0.0011 |
| -2.04 | 1.977 | 0.1169 | -0.0413 | -0.0539 | 2.649 | 0.0257 | 0.0896 | 0.0010 |
| -1.97 | 1.989 | 0.1139 | -0.0336 | -0.0511 | 2.637 | 0.0263 | 0.0904 | 0.0009 |
| -1.89 | 2.001 | 0.1110 | -0.0262 | -0.0485 | 2.625 | 0.0270 | 0.0911 | 0.0007 |
| -1.82 | 2.012 | 0.1082 | -0.0191 | -0.0459 | 2.613 | 0.0276 | 0.0918 | 0.0006 |
| -1.75 | 2.024 | 0.1054 | -0.0123 | -0.0435 | 2.602 | 0.0283 | 0.0924 | 0.0004 |
| -1.68 | 2.036 | 0.1027 | -0.0058 | -0.0411 | 2.590 | 0.0291 | 0.0930 | 0.0003 |
| -1.60 | 2.048 | 0.1000 | 0.0004 | -0.0389 | 2.578 | 0.0298 | 0.0934 | 0.0001 |
| -1.53 | 2.060 | 0.0974 | 0.0064 | -0.0367 | 2.566 | 0.0306 | 0.0937 | -0.0001 |
| -1.46 | 2.072 | 0.0949 | 0.0120 | -0.0347 | 2.554 | 0.0315 | 0.0940 | -0.0004 |
| -1.38 | 2.084 | 0.0924 | 0.0174 | -0.0328 | 2.542 | 0.0323 | 0.0941 | -0.0006 |
| -1.31 | 2.096 | 0.0899 | 0.0225 | -0.0309 | 2.530 | 0.0332 | 0.0941 | -0.0009 |
| -1.24 | 2.108 | 0.0876 | 0.0274 | -0.0292 | 2.518 | 0.0341 | 0.0940 | -0.0011 |
| -1.17 | 2.121 | 0.0853 | 0.0320 | -0.0275 | 2.506 | 0.0351 | 0.0938 | -0.0014 |
| -1.09 | 2.133 | 0.0830 | 0.0363 | -0.0259 | 2.494 | 0.0361 | 0.0935 | -0.0018 |
| -1.02 | 2.145 | 0.0808 | 0.0404 | -0.0244 | 2.482 | 0.0372 | 0.0931 | -0.0021 |
| -0.95 | 2.157 | 0.0787 | 0.0443 | -0.0230 | 2.470 | 0.0383 | 0.0925 | -0.0025 |
| -0.87 | 2.169 | 0.0766 | 0.0479 | -0.0216 | 2.458 | 0.0394 | 0.0919 | -0.0029 |
| -0.80 | 2.181 | 0.0746 | 0.0513 | -0.0203 | 2.446 | 0.0406 | 0.0911 | -0.0033 |
| -0.73 | 2.193 | 0.0726 | 0.0546 | -0.0191 | 2.434 | 0.0417 | 0.0903 | -0.0038 |
| -0.66 | 2.205 | 0.0707 | 0.0576 | -0.0179 | 2.422 | 0.0430 | 0.0893 | -0.0042 |
| -0.58 | 2.217 | 0.0688 | 0.0604 | -0.0168 | 2.410 | 0.0442 | 0.0883 | -0.0047 |
| -0.51 | 2.229 | 0.0670 | 0.0630 | -0.0158 | 2.397 | 0.0455 | 0.0872 | -0.0053 |
| -0.44 | 2.241 | 0.0652 | 0.0655 | -0.0148 | 2.385 | 0.0468 | 0.0860 | -0.0058 |
| -0.36 | 2.253 | 0.0635 | 0.0679 | -0.0138 | 2.373 | 0.0482 | 0.0848 | -0.0064 |
| -0.29 | 2.265 | 0.0618 | 0.0700 | -0.0129 | 2.361 | 0.0496 | 0.0835 | -0.0070 |
| -0.22 | 2.277 | 0.0601 | 0.0721 | -0.0120 | 2.349 | 0.0510 | 0.0821 | -0.0076 |
| -0.15 | 2.289 | 0.0585 | 0.0740 | -0.0112 | 2.337 | 0.0524 | 0.0807 | -0.0083 |
| -0.07 | 2.301 | 0.0570 | 0.0758 | -0.0104 | 2.325 | 0.0539 | 0.0791 | -0.0090 |
| 0.00 | 2.313 | 0.0554 | 0.0775 | -0.0097 | 2.313 | 0.0554 | 0.0775 | -0.0097 |

| **BrCH_3_Br** | C-Br_out_ | | | | C-Br_in_ | | | |
| --- | --- | --- | --- | --- | --- | --- | --- | --- |
| IRC | C-Br dist. (Å) | ρ_BCP_ | ∇^2^ρ_BCP_ | H_BCP_ | C-Br dist. (Å) | ρ_BCP_ | ∇^2^ρ_BCP_ | H_BCP_ |
| -6.48 | 1.986 | 0.1332 | -0.1064 | -0.0689 | 3.213 | 0.0114 | 0.0392 | 0.0015 |
| -6.41 | 1.986 | 0.1333 | -0.1065 | -0.0690 | 3.207 | 0.0115 | 0.0397 | 0.0015 |
| -6.34 | 1.986 | 0.1332 | -0.1063 | -0.0689 | 3.201 | 0.0117 | 0.0401 | 0.0015 |
| -6.26 | 1.986 | 0.1333 | -0.1063 | -0.0690 | 3.196 | 0.0118 | 0.0405 | 0.0015 |
| -6.19 | 1.986 | 0.1332 | -0.1063 | -0.0690 | 3.190 | 0.0119 | 0.0409 | 0.0015 |
| -6.12 | 1.986 | 0.1333 | -0.1062 | -0.0690 | 3.184 | 0.0120 | 0.0414 | 0.0015 |
| -6.05 | 1.986 | 0.1332 | -0.1062 | -0.0690 | 3.179 | 0.0121 | 0.0418 | 0.0015 |
| -5.97 | 1.986 | 0.1333 | -0.1061 | -0.0690 | 3.173 | 0.0122 | 0.0422 | 0.0015 |
| -5.90 | 1.986 | 0.1333 | -0.1061 | -0.0690 | 3.167 | 0.0123 | 0.0427 | 0.0015 |
| -5.83 | 1.986 | 0.1333 | -0.1061 | -0.0690 | 3.162 | 0.0125 | 0.0431 | 0.0015 |
| -5.75 | 1.986 | 0.1333 | -0.1060 | -0.0690 | 3.156 | 0.0126 | 0.0436 | 0.0015 |
| -5.68 | 1.986 | 0.1333 | -0.1060 | -0.0691 | 3.151 | 0.0127 | 0.0440 | 0.0015 |
| -5.61 | 1.986 | 0.1333 | -0.1059 | -0.0691 | 3.145 | 0.0128 | 0.0445 | 0.0015 |
| -5.54 | 1.985 | 0.1333 | -0.1059 | -0.0691 | 3.139 | 0.0129 | 0.0450 | 0.0015 |
| -5.46 | 1.985 | 0.1333 | -0.1058 | -0.0691 | 3.134 | 0.0131 | 0.0454 | 0.0015 |
| -5.39 | 1.985 | 0.1333 | -0.1058 | -0.0691 | 3.128 | 0.0132 | 0.0459 | 0.0015 |
| -5.32 | 1.985 | 0.1333 | -0.1057 | -0.0691 | 3.122 | 0.0133 | 0.0464 | 0.0015 |
| -5.24 | 1.985 | 0.1334 | -0.1058 | -0.0692 | 3.117 | 0.0134 | 0.0468 | 0.0015 |
| -5.17 | 1.985 | 0.1333 | -0.1057 | -0.0692 | 3.111 | 0.0136 | 0.0473 | 0.0015 |
| -5.10 | 1.985 | 0.1334 | -0.1058 | -0.0693 | 3.106 | 0.0137 | 0.0478 | 0.0015 |
| -5.03 | 1.985 | 0.1334 | -0.1056 | -0.0692 | 3.100 | 0.0138 | 0.0483 | 0.0015 |
| -4.95 | 1.984 | 0.1335 | -0.1057 | -0.0693 | 3.095 | 0.0139 | 0.0488 | 0.0015 |
| -4.88 | 1.984 | 0.1336 | -0.1060 | -0.0695 | 3.089 | 0.0141 | 0.0492 | 0.0015 |
| -4.81 | 1.984 | 0.1335 | -0.1058 | -0.0694 | 3.083 | 0.0142 | 0.0498 | 0.0015 |
| -4.73 | 1.984 | 0.1335 | -0.1057 | -0.0694 | 3.078 | 0.0144 | 0.0503 | 0.0015 |
| -4.66 | 1.984 | 0.1335 | -0.1057 | -0.0694 | 3.072 | 0.0145 | 0.0508 | 0.0015 |
| -4.59 | 1.984 | 0.1336 | -0.1057 | -0.0695 | 3.067 | 0.0146 | 0.0513 | 0.0015 |
| -4.52 | 1.983 | 0.1336 | -0.1057 | -0.0695 | 3.061 | 0.0148 | 0.0518 | 0.0015 |
| -4.44 | 1.983 | 0.1336 | -0.1057 | -0.0696 | 3.056 | 0.0149 | 0.0524 | 0.0015 |
| -4.37 | 1.983 | 0.1337 | -0.1057 | -0.0696 | 3.050 | 0.0150 | 0.0529 | 0.0015 |
| -4.30 | 1.983 | 0.1337 | -0.1058 | -0.0697 | 3.045 | 0.0152 | 0.0534 | 0.0015 |
| -4.22 | 1.983 | 0.1338 | -0.1058 | -0.0697 | 3.039 | 0.0153 | 0.0539 | 0.0015 |
| -4.15 | 1.982 | 0.1338 | -0.1058 | -0.0698 | 3.034 | 0.0155 | 0.0545 | 0.0015 |
| -4.08 | 1.982 | 0.1340 | -0.1060 | -0.0699 | 3.028 | 0.0156 | 0.0550 | 0.0015 |
| -4.01 | 1.982 | 0.1340 | -0.1060 | -0.0699 | 3.023 | 0.0158 | 0.0555 | 0.0015 |
| -3.93 | 1.982 | 0.1340 | -0.1059 | -0.0699 | 3.017 | 0.0159 | 0.0561 | 0.0015 |
| -3.86 | 1.981 | 0.1340 | -0.1059 | -0.0700 | 3.012 | 0.0161 | 0.0566 | 0.0015 |
| -3.79 | 1.981 | 0.1340 | -0.1059 | -0.0700 | 3.006 | 0.0162 | 0.0572 | 0.0015 |
| -3.71 | 1.981 | 0.1341 | -0.1059 | -0.0701 | 3.000 | 0.0164 | 0.0578 | 0.0015 |
| -3.64 | 1.981 | 0.1340 | -0.1057 | -0.0700 | 2.995 | 0.0165 | 0.0584 | 0.0015 |
| -3.57 | 1.981 | 0.1340 | -0.1054 | -0.0700 | 2.989 | 0.0167 | 0.0589 | 0.0015 |
| -3.50 | 1.982 | 0.1338 | -0.1049 | -0.0699 | 2.983 | 0.0169 | 0.0595 | 0.0015 |
| -3.42 | 1.982 | 0.1336 | -0.1045 | -0.0697 | 2.977 | 0.0171 | 0.0603 | 0.0015 |
| -3.35 | 1.986 | 0.1327 | -0.1018 | -0.0688 | 2.968 | 0.0173 | 0.0608 | 0.0015 |
| -3.28 | 1.989 | 0.1319 | -0.0998 | -0.0680 | 2.960 | 0.0176 | 0.0617 | 0.0015 |
| -3.20 | 1.994 | 0.1306 | -0.0964 | -0.0667 | 2.951 | 0.0178 | 0.0625 | 0.0014 |
| -3.13 | 2.000 | 0.1290 | -0.0922 | -0.0652 | 2.940 | 0.0182 | 0.0634 | 0.0014 |
| -3.06 | 2.008 | 0.1271 | -0.0873 | -0.0633 | 2.930 | 0.0185 | 0.0643 | 0.0014 |
| -2.99 | 2.017 | 0.1249 | -0.0820 | -0.0613 | 2.919 | 0.0189 | 0.0651 | 0.0013 |
| -2.91 | 2.026 | 0.1227 | -0.0764 | -0.0592 | 2.908 | 0.0193 | 0.0660 | 0.0013 |
| -2.84 | 2.036 | 0.1204 | -0.0708 | -0.0571 | 2.896 | 0.0197 | 0.0668 | 0.0012 |
| -2.77 | 2.046 | 0.1180 | -0.0652 | -0.0550 | 2.885 | 0.0201 | 0.0676 | 0.0012 |
| -2.70 | 2.056 | 0.1157 | -0.0596 | -0.0529 | 2.874 | 0.0205 | 0.0684 | 0.0011 |
| -2.62 | 2.066 | 0.1133 | -0.0541 | -0.0508 | 2.863 | 0.0209 | 0.0691 | 0.0011 |
| -2.55 | 2.077 | 0.1110 | -0.0488 | -0.0488 | 2.852 | 0.0214 | 0.0698 | 0.0010 |
| -2.48 | 2.087 | 0.1087 | -0.0435 | -0.0469 | 2.841 | 0.0218 | 0.0705 | 0.0009 |
| -2.40 | 2.098 | 0.1064 | -0.0384 | -0.0450 | 2.830 | 0.0223 | 0.0712 | 0.0008 |
| -2.33 | 2.109 | 0.1042 | -0.0334 | -0.0431 | 2.819 | 0.0228 | 0.0718 | 0.0008 |
| -2.26 | 2.119 | 0.1019 | -0.0286 | -0.0413 | 2.807 | 0.0233 | 0.0724 | 0.0007 |
| -2.19 | 2.130 | 0.0997 | -0.0239 | -0.0396 | 2.796 | 0.0238 | 0.0729 | 0.0006 |
| -2.11 | 2.141 | 0.0976 | -0.0193 | -0.0379 | 2.785 | 0.0243 | 0.0734 | 0.0005 |
| -2.04 | 2.152 | 0.0955 | -0.0149 | -0.0363 | 2.774 | 0.0249 | 0.0738 | 0.0003 |
| -1.97 | 2.163 | 0.0934 | -0.0106 | -0.0347 | 2.763 | 0.0254 | 0.0742 | 0.0002 |
| -1.89 | 2.174 | 0.0913 | -0.0064 | -0.0332 | 2.752 | 0.0260 | 0.0745 | 0.0001 |
| -1.82 | 2.185 | 0.0893 | -0.0024 | -0.0317 | 2.741 | 0.0266 | 0.0748 | -0.0001 |
| -1.75 | 2.196 | 0.0873 | 0.0015 | -0.0303 | 2.730 | 0.0273 | 0.0750 | -0.0002 |
| -1.68 | 2.207 | 0.0854 | 0.0052 | -0.0290 | 2.719 | 0.0279 | 0.0751 | -0.0004 |
| -1.60 | 2.218 | 0.0834 | 0.0088 | -0.0276 | 2.708 | 0.0286 | 0.0752 | -0.0006 |
| -1.53 | 2.229 | 0.0816 | 0.0123 | -0.0264 | 2.697 | 0.0293 | 0.0752 | -0.0008 |
| -1.46 | 2.240 | 0.0797 | 0.0157 | -0.0251 | 2.686 | 0.0300 | 0.0751 | -0.0010 |
| -1.38 | 2.251 | 0.0779 | 0.0190 | -0.0240 | 2.675 | 0.0308 | 0.0750 | -0.0012 |
| -1.31 | 2.262 | 0.0761 | 0.0221 | -0.0228 | 2.663 | 0.0316 | 0.0747 | -0.0014 |
| -1.24 | 2.273 | 0.0744 | 0.0251 | -0.0217 | 2.652 | 0.0324 | 0.0744 | -0.0017 |
| -1.17 | 2.284 | 0.0727 | 0.0280 | -0.0207 | 2.641 | 0.0332 | 0.0740 | -0.0020 |
| -1.09 | 2.295 | 0.0710 | 0.0307 | -0.0197 | 2.630 | 0.0341 | 0.0735 | -0.0022 |
| -1.02 | 2.307 | 0.0694 | 0.0334 | -0.0187 | 2.619 | 0.0350 | 0.0729 | -0.0025 |
| -0.95 | 2.318 | 0.0678 | 0.0359 | -0.0178 | 2.608 | 0.0359 | 0.0723 | -0.0029 |
| -0.87 | 2.329 | 0.0662 | 0.0383 | -0.0169 | 2.597 | 0.0369 | 0.0716 | -0.0032 |
| -0.80 | 2.340 | 0.0647 | 0.0406 | -0.0160 | 2.585 | 0.0378 | 0.0709 | -0.0035 |
| -0.73 | 2.351 | 0.0632 | 0.0427 | -0.0152 | 2.574 | 0.0388 | 0.0701 | -0.0039 |
| -0.66 | 2.362 | 0.0617 | 0.0448 | -0.0144 | 2.563 | 0.0398 | 0.0692 | -0.0043 |
| -0.58 | 2.373 | 0.0603 | 0.0468 | -0.0136 | 2.552 | 0.0408 | 0.0683 | -0.0047 |
| -0.51 | 2.384 | 0.0589 | 0.0487 | -0.0129 | 2.541 | 0.0419 | 0.0674 | -0.0051 |
| -0.44 | 2.396 | 0.0575 | 0.0505 | -0.0122 | 2.529 | 0.0430 | 0.0664 | -0.0055 |
| -0.36 | 2.407 | 0.0562 | 0.0522 | -0.0115 | 2.518 | 0.0441 | 0.0654 | -0.0060 |
| -0.29 | 2.418 | 0.0548 | 0.0538 | -0.0108 | 2.507 | 0.0452 | 0.0643 | -0.0064 |
| -0.22 | 2.429 | 0.0535 | 0.0553 | -0.0102 | 2.496 | 0.0463 | 0.0632 | -0.0069 |
| -0.15 | 2.440 | 0.0523 | 0.0568 | -0.0096 | 2.485 | 0.0475 | 0.0620 | -0.0074 |
| -0.07 | 2.451 | 0.0510 | 0.0582 | -0.0090 | 2.474 | 0.0486 | 0.0608 | -0.0079 |
| 0.00 | 2.462 | 0.0498 | 0.0595 | -0.0085 | 2.462 | 0.0498 | 0.0595 | -0.0085 |

| **ICH_3_I** | C-I_out_ | | | | C-I_in_ | | | |
| --- | --- | --- | --- | --- | --- | --- | --- | --- |
| IRC | C-I  dist. (Å) | ρ_BCP_ | ∇^2^ρ_BCP_ | H_BCP_ | C-I  dist. (Å) | ρ_BCP_ | ∇^2^ρ_BCP_ | H_BCP_ |
| -7.65 | 2.172 | 0.1129 | -0.0777 | -0.0554 | 3.423 | 0.0105 | 0.0322 | 0.0012 |
| -7.58 | 2.172 | 0.1129 | -0.0777 | -0.0554 | 3.418 | 0.0106 | 0.0325 | 0.0012 |
| -7.50 | 2.172 | 0.1129 | -0.0777 | -0.0554 | 3.414 | 0.0107 | 0.0327 | 0.0012 |
| -7.43 | 2.172 | 0.1129 | -0.0777 | -0.0554 | 3.410 | 0.0108 | 0.0330 | 0.0012 |
| -7.36 | 2.172 | 0.1129 | -0.0777 | -0.0554 | 3.405 | 0.0108 | 0.0332 | 0.0012 |
| -7.28 | 2.172 | 0.1130 | -0.0777 | -0.0554 | 3.401 | 0.0109 | 0.0335 | 0.0012 |
| -7.21 | 2.172 | 0.1130 | -0.0777 | -0.0554 | 3.396 | 0.0110 | 0.0338 | 0.0012 |
| -7.14 | 2.171 | 0.1130 | -0.0777 | -0.0555 | 3.392 | 0.0111 | 0.0340 | 0.0012 |
| -7.07 | 2.171 | 0.1130 | -0.0777 | -0.0555 | 3.388 | 0.0112 | 0.0343 | 0.0012 |
| -6.99 | 2.171 | 0.1130 | -0.0777 | -0.0555 | 3.383 | 0.0112 | 0.0345 | 0.0012 |
| -6.92 | 2.171 | 0.1130 | -0.0777 | -0.0555 | 3.379 | 0.0113 | 0.0348 | 0.0012 |
| -6.85 | 2.171 | 0.1130 | -0.0777 | -0.0555 | 3.374 | 0.0114 | 0.0351 | 0.0012 |
| -6.77 | 2.171 | 0.1131 | -0.0777 | -0.0555 | 3.370 | 0.0115 | 0.0354 | 0.0012 |
| -6.70 | 2.171 | 0.1131 | -0.0777 | -0.0555 | 3.366 | 0.0115 | 0.0356 | 0.0012 |
| -6.63 | 2.171 | 0.1131 | -0.0777 | -0.0556 | 3.361 | 0.0116 | 0.0359 | 0.0012 |
| -6.56 | 2.170 | 0.1131 | -0.0777 | -0.0556 | 3.357 | 0.0117 | 0.0362 | 0.0012 |
| -6.48 | 2.170 | 0.1131 | -0.0777 | -0.0556 | 3.353 | 0.0118 | 0.0365 | 0.0012 |
| -6.41 | 2.170 | 0.1132 | -0.0777 | -0.0556 | 3.348 | 0.0119 | 0.0367 | 0.0012 |
| -6.34 | 2.170 | 0.1132 | -0.0777 | -0.0556 | 3.344 | 0.0119 | 0.0370 | 0.0012 |
| -6.26 | 2.170 | 0.1132 | -0.0777 | -0.0556 | 3.340 | 0.0120 | 0.0373 | 0.0012 |
| -6.19 | 2.170 | 0.1132 | -0.0777 | -0.0557 | 3.335 | 0.0121 | 0.0376 | 0.0012 |
| -6.12 | 2.170 | 0.1132 | -0.0777 | -0.0557 | 3.331 | 0.0122 | 0.0379 | 0.0012 |
| -6.05 | 2.169 | 0.1133 | -0.0777 | -0.0557 | 3.326 | 0.0123 | 0.0382 | 0.0012 |
| -5.97 | 2.169 | 0.1133 | -0.0777 | -0.0557 | 3.322 | 0.0124 | 0.0385 | 0.0012 |
| -5.90 | 2.169 | 0.1133 | -0.0777 | -0.0557 | 3.318 | 0.0125 | 0.0387 | 0.0012 |
| -5.83 | 2.169 | 0.1133 | -0.0777 | -0.0558 | 3.313 | 0.0125 | 0.0390 | 0.0012 |
| -5.75 | 2.169 | 0.1134 | -0.0777 | -0.0558 | 3.309 | 0.0126 | 0.0393 | 0.0012 |
| -5.68 | 2.169 | 0.1134 | -0.0777 | -0.0558 | 3.305 | 0.0127 | 0.0396 | 0.0012 |
| -5.61 | 2.168 | 0.1134 | -0.0777 | -0.0558 | 3.300 | 0.0128 | 0.0399 | 0.0012 |
| -5.54 | 2.168 | 0.1134 | -0.0777 | -0.0559 | 3.296 | 0.0129 | 0.0402 | 0.0012 |
| -5.46 | 2.168 | 0.1135 | -0.0777 | -0.0559 | 3.292 | 0.0130 | 0.0405 | 0.0012 |
| -5.39 | 2.168 | 0.1135 | -0.0777 | -0.0559 | 3.287 | 0.0131 | 0.0408 | 0.0012 |
| -5.32 | 2.168 | 0.1135 | -0.0777 | -0.0560 | 3.283 | 0.0132 | 0.0411 | 0.0012 |
| -5.24 | 2.167 | 0.1136 | -0.0777 | -0.0560 | 3.279 | 0.0132 | 0.0414 | 0.0012 |
| -5.17 | 2.167 | 0.1136 | -0.0777 | -0.0560 | 3.274 | 0.0133 | 0.0417 | 0.0012 |
| -5.10 | 2.167 | 0.1136 | -0.0778 | -0.0560 | 3.270 | 0.0134 | 0.0421 | 0.0012 |
| -5.03 | 2.167 | 0.1137 | -0.0778 | -0.0561 | 3.266 | 0.0135 | 0.0424 | 0.0012 |
| -4.95 | 2.167 | 0.1137 | -0.0778 | -0.0561 | 3.262 | 0.0136 | 0.0427 | 0.0012 |
| -4.88 | 2.166 | 0.1137 | -0.0778 | -0.0561 | 3.257 | 0.0137 | 0.0430 | 0.0012 |
| -4.81 | 2.166 | 0.1138 | -0.0778 | -0.0562 | 3.253 | 0.0138 | 0.0433 | 0.0012 |
| -4.73 | 2.166 | 0.1138 | -0.0778 | -0.0562 | 3.249 | 0.0139 | 0.0436 | 0.0012 |
| -4.66 | 2.166 | 0.1139 | -0.0779 | -0.0563 | 3.245 | 0.0140 | 0.0439 | 0.0012 |
| -4.59 | 2.166 | 0.1139 | -0.0778 | -0.0563 | 3.240 | 0.0141 | 0.0443 | 0.0012 |
| -4.52 | 2.165 | 0.1140 | -0.0779 | -0.0563 | 3.236 | 0.0142 | 0.0446 | 0.0012 |
| -4.44 | 2.165 | 0.1140 | -0.0780 | -0.0564 | 3.232 | 0.0143 | 0.0449 | 0.0012 |
| -4.37 | 2.165 | 0.1140 | -0.0779 | -0.0564 | 3.227 | 0.0144 | 0.0452 | 0.0012 |
| -4.30 | 2.164 | 0.1141 | -0.0779 | -0.0564 | 3.223 | 0.0145 | 0.0456 | 0.0012 |
| -4.22 | 2.164 | 0.1141 | -0.0779 | -0.0565 | 3.219 | 0.0146 | 0.0459 | 0.0012 |
| -4.15 | 2.164 | 0.1142 | -0.0780 | -0.0565 | 3.215 | 0.0147 | 0.0462 | 0.0012 |
| -4.08 | 2.164 | 0.1142 | -0.0780 | -0.0566 | 3.210 | 0.0148 | 0.0465 | 0.0012 |
| -4.01 | 2.163 | 0.1142 | -0.0780 | -0.0566 | 3.206 | 0.0149 | 0.0469 | 0.0012 |
| -3.93 | 2.163 | 0.1142 | -0.0780 | -0.0566 | 3.202 | 0.0150 | 0.0473 | 0.0012 |
| -3.86 | 2.163 | 0.1142 | -0.0779 | -0.0566 | 3.197 | 0.0151 | 0.0476 | 0.0012 |
| -3.79 | 2.163 | 0.1143 | -0.0779 | -0.0567 | 3.193 | 0.0152 | 0.0480 | 0.0012 |
| -3.71 | 2.164 | 0.1141 | -0.0776 | -0.0564 | 3.188 | 0.0153 | 0.0483 | 0.0012 |
| -3.64 | 2.164 | 0.1140 | -0.0775 | -0.0563 | 3.183 | 0.0155 | 0.0488 | 0.0012 |
| -3.57 | 2.166 | 0.1136 | -0.0770 | -0.0560 | 3.177 | 0.0156 | 0.0492 | 0.0012 |
| -3.50 | 2.169 | 0.1130 | -0.0763 | -0.0555 | 3.170 | 0.0158 | 0.0497 | 0.0011 |
| -3.42 | 2.173 | 0.1122 | -0.0752 | -0.0547 | 3.162 | 0.0160 | 0.0502 | 0.0011 |
| -3.35 | 2.180 | 0.1111 | -0.0735 | -0.0536 | 3.153 | 0.0162 | 0.0508 | 0.0011 |
| -3.28 | 2.187 | 0.1096 | -0.0716 | -0.0522 | 3.143 | 0.0165 | 0.0514 | 0.0011 |
| -3.20 | 2.196 | 0.1080 | -0.0691 | -0.0507 | 3.132 | 0.0168 | 0.0520 | 0.0011 |
| -3.13 | 2.205 | 0.1064 | -0.0665 | -0.0492 | 3.122 | 0.0171 | 0.0526 | 0.0010 |
| -3.06 | 2.214 | 0.1046 | -0.0637 | -0.0476 | 3.111 | 0.0174 | 0.0532 | 0.0010 |
| -2.99 | 2.224 | 0.1029 | -0.0607 | -0.0461 | 3.100 | 0.0178 | 0.0538 | 0.0010 |
| -2.91 | 2.234 | 0.1011 | -0.0576 | -0.0445 | 3.090 | 0.0181 | 0.0544 | 0.0009 |
| -2.84 | 2.244 | 0.0993 | -0.0544 | -0.0430 | 3.079 | 0.0185 | 0.0549 | 0.0009 |
| -2.77 | 2.254 | 0.0976 | -0.0512 | -0.0414 | 3.068 | 0.0188 | 0.0554 | 0.0008 |
| -2.70 | 2.264 | 0.0958 | -0.0479 | -0.0400 | 3.058 | 0.0192 | 0.0559 | 0.0008 |
| -2.62 | 2.275 | 0.0941 | -0.0446 | -0.0385 | 3.047 | 0.0196 | 0.0564 | 0.0007 |
| -2.55 | 2.285 | 0.0923 | -0.0413 | -0.0371 | 3.036 | 0.0199 | 0.0568 | 0.0007 |
| -2.48 | 2.296 | 0.0906 | -0.0380 | -0.0357 | 3.026 | 0.0203 | 0.0572 | 0.0006 |
| -2.40 | 2.306 | 0.0889 | -0.0347 | -0.0344 | 3.015 | 0.0208 | 0.0576 | 0.0005 |
| -2.33 | 2.317 | 0.0872 | -0.0314 | -0.0331 | 3.005 | 0.0212 | 0.0580 | 0.0004 |
| -2.26 | 2.327 | 0.0856 | -0.0281 | -0.0318 | 2.994 | 0.0216 | 0.0583 | 0.0004 |
| -2.19 | 2.338 | 0.0839 | -0.0249 | -0.0306 | 2.983 | 0.0221 | 0.0586 | 0.0003 |
| -2.11 | 2.348 | 0.0823 | -0.0217 | -0.0294 | 2.973 | 0.0225 | 0.0588 | 0.0002 |
| -2.04 | 2.359 | 0.0807 | -0.0186 | -0.0282 | 2.962 | 0.0230 | 0.0590 | 0.0001 |
| -1.97 | 2.369 | 0.0791 | -0.0155 | -0.0271 | 2.951 | 0.0235 | 0.0592 | -0.0001 |
| -1.89 | 2.380 | 0.0776 | -0.0125 | -0.0260 | 2.941 | 0.0241 | 0.0593 | -0.0002 |
| -1.82 | 2.391 | 0.0760 | -0.0095 | -0.0249 | 2.930 | 0.0246 | 0.0593 | -0.0003 |
| -1.75 | 2.401 | 0.0745 | -0.0066 | -0.0239 | 2.919 | 0.0252 | 0.0593 | -0.0004 |
| -1.68 | 2.412 | 0.0730 | -0.0037 | -0.0229 | 2.908 | 0.0257 | 0.0592 | -0.0006 |
| -1.60 | 2.423 | 0.0715 | -0.0009 | -0.0219 | 2.898 | 0.0263 | 0.0591 | -0.0008 |
| -1.53 | 2.433 | 0.0700 | 0.0018 | -0.0210 | 2.887 | 0.0270 | 0.0589 | -0.0009 |
| -1.46 | 2.444 | 0.0686 | 0.0045 | -0.0201 | 2.876 | 0.0276 | 0.0586 | -0.0011 |
| -1.38 | 2.455 | 0.0672 | 0.0071 | -0.0192 | 2.865 | 0.0283 | 0.0583 | -0.0013 |
| -1.31 | 2.466 | 0.0658 | 0.0096 | -0.0183 | 2.855 | 0.0290 | 0.0579 | -0.0015 |
| -1.24 | 2.477 | 0.0644 | 0.0121 | -0.0175 | 2.844 | 0.0297 | 0.0574 | -0.0017 |
| -1.17 | 2.487 | 0.0630 | 0.0144 | -0.0167 | 2.833 | 0.0304 | 0.0569 | -0.0019 |
| -1.09 | 2.498 | 0.0617 | 0.0167 | -0.0159 | 2.822 | 0.0312 | 0.0563 | -0.0022 |
| -1.02 | 2.509 | 0.0604 | 0.0190 | -0.0152 | 2.811 | 0.0319 | 0.0556 | -0.0024 |
| -0.95 | 2.520 | 0.0591 | 0.0211 | -0.0145 | 2.801 | 0.0327 | 0.0549 | -0.0027 |
| -0.87 | 2.530 | 0.0578 | 0.0232 | -0.0138 | 2.790 | 0.0335 | 0.0542 | -0.0030 |
| -0.80 | 2.541 | 0.0566 | 0.0251 | -0.0131 | 2.779 | 0.0343 | 0.0534 | -0.0033 |
| -0.73 | 2.552 | 0.0554 | 0.0270 | -0.0125 | 2.768 | 0.0352 | 0.0526 | -0.0036 |
| -0.66 | 2.563 | 0.0542 | 0.0289 | -0.0119 | 2.757 | 0.0360 | 0.0517 | -0.0039 |
| -0.58 | 2.574 | 0.0530 | 0.0306 | -0.0113 | 2.746 | 0.0369 | 0.0508 | -0.0042 |
| -0.51 | 2.585 | 0.0519 | 0.0323 | -0.0107 | 2.736 | 0.0378 | 0.0499 | -0.0045 |
| -0.44 | 2.595 | 0.0507 | 0.0339 | -0.0101 | 2.725 | 0.0387 | 0.0489 | -0.0049 |
| -0.36 | 2.606 | 0.0496 | 0.0355 | -0.0096 | 2.714 | 0.0396 | 0.0479 | -0.0052 |
| -0.29 | 2.617 | 0.0485 | 0.0369 | -0.0091 | 2.703 | 0.0405 | 0.0468 | -0.0056 |
| -0.22 | 2.628 | 0.0475 | 0.0384 | -0.0086 | 2.692 | 0.0415 | 0.0457 | -0.0060 |
| -0.15 | 2.639 | 0.0464 | 0.0397 | -0.0081 | 2.681 | 0.0425 | 0.0446 | -0.0064 |
| -0.07 | 2.649 | 0.0454 | 0.0410 | -0.0077 | 2.671 | 0.0434 | 0.0435 | -0.0068 |
| 0.00 | 2.660 | 0.0444 | 0.0423 | -0.0072 | 2.660 | 0.0444 | 0.0423 | -0.0072 |

**Table S3.** The Relative Energy Gradient (REG) and Pearson coefficient (R) as calculated by the program ANANKE. Standard analysis.

|  | **F···CH_3_F** | | | **Cl···CH_3_Cl** | | | **Br···CH_3_Br** | | | **I···CH_3_I** | | |
| --- | --- | --- | --- | --- | --- | --- | --- | --- | --- | --- | --- | --- |
| Rank | REG | R | IQA_term | REG | R | IQA_term | REG | R | IQA_term | REG | R | IQA_term |
| 1 | 5.6 | 0.99 | Vcl_C_F(out) | 6.3 | 1.00 | Vxc_C_Cl(out) | 7.8 | 0.99 | Vxc_C_Br(out) | 7.7 | 0.99 | Vxc_C_I(out) |
| 2 | 3.7 | 0.98 | Vxc_C_F(out) | 1.5 | 0.99 | Eintra_Cl(in) | 2.1 | 0.99 | Eintra_Br(in) | 2.4 | 0.99 | Eintra_I(in) |
| 3 | 1.2 | 0.98 | Eintra_F(in) | 0.7 | 1.00 | Vcl_Cl(in)_Cl(out) | 1.3 | 0.99 | Vcl_Br(in)_Br(out) | 1.8 | 0.99 | Vcl_I(in)_I(out) |
| 4 | 0.1 | 0.96 | Vcl_h3_F(out) | 0.3 | 1.00 | Vcl_C_h5 | 0.5 | 0.99 | Vcl_C_h3 | 0.6 | 0.99 | Vcl_C_h5 |
| 5 | 0.1 | 0.96 | Vcl_h4_F(out) | 0.3 | 1.00 | Vcl_C_h3 | 0.5 | 0.99 | Vcl_C_h5 | 0.6 | 0.99 | Vcl_C_h4 |
| 6 | 0.1 | 0.96 | Vcl_h5_F(out) | 0.3 | 1.00 | Vcl_C_h4 | 0.5 | 0.99 | Vcl_C_h4 | 0.6 | 0.99 | Vcl_C_h3 |
| 7 | 0.1 | 0.79 | Eintra_h4 | 0.2 | 0.97 | Eintra_h4 | 0.2 | 0.97 | Eintra_h4 | 0.2 | 0.63 | Vcl_C_I(out) |
| 8 | 0.1 | 0.80 | Eintra_h3 | 0.2 | 0.97 | Eintra_h5 | 0.2 | 0.97 | Eintra_h5 | 0.2 | 0.97 | Eintra_h3 |
| 9 | 0.1 | 0.80 | Eintra_h5 | 0.2 | 0.97 | Eintra_h3 | 0.2 | 0.98 | Eintra_h3 | 0.2 | 0.97 | Eintra_h4 |
| 10 | 0.0 | 0.98 | Vxc_h4_h5 | 0.0 | 0.80 | Vxc_h4_Cl(out) | 0.0 | 0.97 | Vcl_h4_h5 | 0.2 | 0.98 | Eintra_h5 |
| 11 | 0.0 | 0.98 | Vxc_h3_h5 | 0.0 | 0.80 | Vxc_h5_Cl(out) | 0.0 | 0.97 | Vcl_h3_h4 | 0.1 | 0.58 | Vcl_I(in)_h4 |
| 12 | 0.0 | 0.98 | Vxc_h3_h4 | 0.0 | 0.79 | Vxc_h3_Cl(out) | 0.0 | 0.97 | Vcl_h3_h5 | 0.1 | 0.58 | Vcl_I(in)_h5 |
| 13 | 0.0 | 0.76 | Vxc_h5_F(out) | 0.0 | 0.96 | Vcl_h4_h5 | 0.0 | 0.68 | Vxc_h4_Br(out) | 0.0 | 0.98 | Vcl_h3_h4 |
| 14 | 0.0 | 0.76 | Vxc_h4_F(out) | 0.0 | 0.96 | Vcl_h3_h4 | 0.0 | 0.68 | Vxc_h5_Br(out) | 0.0 | 0.98 | Vcl_h3_h5 |
| 15 | 0.0 | 0.76 | Vxc_h3_F(out) | 0.0 | 0.96 | Vcl_h3_h5 | 0.0 | 0.66 | Vxc_h3_Br(out) | 0.0 | 0.98 | Vcl_h4_h5 |
| 16 | 0.0 | 0.27 | Vcl_F(in)_h3 | 0.0 | 0.97 | Vxc_h4_h5 | 0.0 | 0.96 | Vxc_h4_h5 | 0.0 | 0.42 | Vcl_I(in)_h3 |
| 17 | 0.0 | 0.26 | Vcl_F(in)_h4 | 0.0 | 0.97 | Vxc_h3_h4 | 0.0 | 0.97 | Vxc_h3_h5 | 0.0 | 0.97 | Vxc_h3_h5 |
| 18 | 0.0 | 0.26 | Vcl_F(in)_h5 | 0.0 | 0.97 | Vxc_h3_h5 | 0.0 | 0.97 | Vxc_h3_h4 | 0.0 | 0.97 | Vxc_h3_h4 |
| 19 | 0.0 | -0.06 | Vcl_C_h3 | 0.0 | -0.45 | Vcl_Cl(in)_h3 | 0.0 | 0.09 | Vcl_Br(in)_h3 | 0.0 | 0.97 | Vxc_h4_h5 |
| 20 | 0.0 | -0.08 | Vcl_C_h5 | 0.0 | -0.47 | Vcl_Cl(in)_h5 | 0.0 | -0.01 | Vcl_Br(in)_h5 | 0.0 | 0.39 | Vxc_h3_I(out) |
| 21 | 0.0 | -0.08 | Vcl_C_h4 | 0.0 | -0.47 | Vcl_Cl(in)_h4 | 0.0 | -0.01 | Vcl_Br(in)_h4 | 0.0 | 0.32 | Vxc_h4_I(out) |
| 22 | 0.0 | -0.93 | Vcl_h4_h5 | -0.1 | -0.51 | Vxc_Cl(in)_h3 | -0.1 | -0.53 | Vxc_Br(in)_h3 | 0.0 | 0.32 | Vxc_h5_I(out) |
| 23 | 0.0 | -0.93 | Vcl_h3_h5 | -0.1 | -0.52 | Vxc_Cl(in)_h5 | -0.1 | -0.55 | Vxc_Br(in)_h5 | 0.0 | -0.13 | Eintra_C |
| 24 | 0.0 | -0.93 | Vcl_h3_h4 | -0.1 | -0.52 | Vxc_Cl(in)_h4 | -0.1 | -0.55 | Vxc_Br(in)_h4 | -0.1 | -0.55 | Vxc_I(in)_h4 |
| 25 | 0.0 | -0.16 | Vcl_C_F(in) | -0.1 | -0.77 | Vcl_C_Cl(in) | -0.1 | -0.35 | Eintra_C | -0.1 | -0.55 | Vxc_I(in)_h5 |
| 26 | -0.1 | -0.56 | Vxc_F(in)_h3 | -0.2 | -0.99 | Vxc_C_h4 | -0.2 | -0.99 | Vxc_C_h4 | -0.1 | -0.61 | Vxc_I(in)_h3 |
| 27 | -0.1 | -0.56 | Vxc_F(in)_h5 | -0.2 | -0.99 | Vxc_C_h5 | -0.2 | -0.99 | Vxc_C_h5 | -0.2 | -0.97 | Vxc_C_h3 |
| 28 | -0.1 | -0.56 | Vxc_F(in)_h4 | -0.2 | -0.99 | Vxc_C_h3 | -0.2 | -0.99 | Vxc_C_h3 | -0.2 | -0.98 | Vxc_C_h4 |
| 29 | -0.1 | -1.00 | Vxc_F(in)_F(out) | -0.2 | -1.00 | Vxc_Cl(in)_Cl(out) | -0.3 | -1.00 | Vxc_Br(in)_Br(out) | -0.2 | -0.98 | Vxc_C_h5 |
| 30 | -0.2 | -0.99 | Vxc_C_h4 | -0.3 | -1.00 | Vcl_h3_Cl(out) | -0.5 | -0.94 | Vcl_C_Br(in) | -0.3 | -1.00 | Vxc_I(in)_I(out) |
| 31 | -0.2 | -0.99 | Vxc_C_h5 | -0.3 | -1.00 | Vcl_h5_Cl(out) | -0.6 | -1.00 | Vcl_h3_Br(out) | -0.8 | -1.00 | Vcl_h3_I(out) |
| 32 | -0.2 | -0.99 | Vxc_C_h3 | -0.3 | -1.00 | Vcl_h4_Cl(out) | -0.6 | -1.00 | Vcl_h5_Br(out) | -0.8 | -1.00 | Vcl_h5_I(out) |
| 33 | -0.4 | -0.93 | Vcl_F(in)_F(out) | -0.3 | -0.84 | Eintra_C | -0.6 | -1.00 | Vcl_h4_Br(out) | -0.8 | -1.00 | Vcl_h4_I(out) |
| 34 | -2.4 | -0.98 | Eintra_C | -1.1 | -0.99 | Vcl_C_Cl(out) | -1.5 | -0.99 | Vcl_C_Br(out) | -1.0 | -0.97 | Vcl_C_I(in) |
| 35 | -3.3 | -0.98 | Vxc_C_F(in) | -2.6 | -1.00 | Eintra_Cl(out) | -3.7 | -0.98 | Vxc_C_Br(in) | -3.7 | -0.98 | Vxc_C_I(in) |
| 36 | -3.4 | -0.99 | Eintra_F(out) | -3.0 | -0.97 | Vxc_C_Cl(in) | -3.8 | -1.00 | Eintra_Br(out) | -5.7 | -0.99 | Eintra_I(out) |

**Table S4.** The Relative Energy Gradient (REG) and Pearson coefficient (R) as calculated by the program ANANKE. Refined analysis (i.e. less coarse-grained than Table S3): (i) breakdown of Intra-atomic energies (Coulomb, exchange and kinetic energy (KE)); (ii) division of reaction barrier into two segments: close segment (ξ > -2.9) and far segment (ξ < -2.9) (but for I ξ = -3.3 is the segment boundary).

| **X = F** | | | | | |
| --- | --- | --- | --- | --- | --- |
| **Close Segment (ξ > -2.9)** | | | **Far Segment (ξ < -2.9)** | | |
| TERM | REG | R | TERM | REG | R |
| Intra_Vcl_F(out) | 4.29 | 1.00 | Intra_ke_F(in) | 12.35 | 0.98 |
| Intra_Vx_F(in) | 3.32 | 0.96 | Intra_ke_C | 3.41 | 0.97 |
| Intra_ke_C | 1.15 | 0.81 | Intra_ke_F(out) | 2.75 | 0.96 |
| Intra_ke_F(in) | 0.98 | 0.71 | Intra_ke_H3 | 2.06 | 0.97 |
| Intra_Vx_F(out) | 0.40 | 0.31 | Intra_ke_H4 | 2.00 | 0.99 |
| Intra_ke_H4 | 0.29 | 0.99 | Intra_ke_H5 | 2.00 | 0.99 |
| Intra_ke_H5 | 0.29 | 0.99 | Intra_Vx_C | 1.46 | 0.99 |
| Intra_ke_H3 | 0.29 | 0.98 | Intra_Vx_F(in) | 0.57 | 1.00 |
| Intra_Vx_H5 | -0.05 | -0.72 | Intra_Vx_H5 | -0.55 | -0.99 |
| Intra_Vx_H3 | -0.05 | -0.72 | Intra_Vx_H4 | -0.56 | -1.00 |
| Intra_Vx_H4 | -0.05 | -0.72 | Intra_Vx_H3 | -0.58 | -0.97 |
| Intra_Vcl_H3 | -0.21 | -0.99 | Intra_Vcl_H4 | -0.90 | -0.99 |
| Intra_Vcl_H4 | -0.22 | -1.00 | Intra_Vcl_H5 | -0.91 | -0.99 |
| Intra_Vcl_H5 | -0.22 | -0.99 | Intra_Vcl_H3 | -0.94 | -0.96 |
| Intra_Vx_C | -1.28 | -0.96 | Intra_Vx_F(out) | -1.34 | -0.96 |
| Intra_Vcl_C | -2.46 | -0.92 | Intra_Vcl_F(out) | -1.74 | -0.97 |
| Intra_Vcl_F(in) | -3.31 | -0.99 | Intra_Vcl_C | -2.95 | -0.97 |
| Intra_ke_F(out) | -8.19 | -0.98 | Intra_Vcl_F(in) | -9.65 | -0.98 |
| **X = Cl** | | | | | |
| **Close Segment (ξ > -2.9)** | | | **Far Segment (ξ < -2.9)** | | |
| TERM | REG | R | TERM | REG | R |
| Intra_Vx_Cl(in) | 4.15 | 0.95 | Intra_ke_Cl(in) | 11.70 | 0.98 |
| Intra_Vcl_Cl(out) | 3.60 | 0.97 | Intra_ke_C | 3.95 | 0.88 |
| Intra_Vcl_C | 1.33 | 0.98 | Intra_ke_Cl(out) | 2.85 | 0.59 |
| Intra_Vx_C | 0.20 | 0.80 | Intra_ke_H5 | 0.99 | 0.93 |
| Intra_Vx_H5 | 0.19 | 0.95 | Intra_ke_H3 | 0.99 | 0.91 |
| Intra_Vx_H4 | 0.19 | 0.94 | Intra_ke_H4 | 0.91 | 0.94 |
| Intra_Vx_H3 | 0.18 | 0.94 | Intra_Vx_Cl(in) | 0.81 | 0.98 |
| Intra_ke_H3 | -0.03 | -0.32 | Intra_Vx_C | 0.23 | 0.76 |
| Intra_ke_H5 | -0.03 | -0.33 | Intra_Vx_H4 | 0.03 | 0.25 |
| Intra_ke_H4 | -0.03 | -0.33 | Intra_Vx_H5 | 0.00 | 0.03 |
| Intra_Vcl_H4 | -0.03 | -0.68 | Intra_Vx_H3 | -0.01 | -0.08 |
| Intra_Vcl_H5 | -0.03 | -0.68 | Intra_Vcl_H4 | -0.45 | -0.92 |
| Intra_Vcl_H3 | -0.03 | -0.69 | Intra_Vcl_H3 | -0.52 | -0.88 |
| Intra_ke_Cl(in) | -0.85 | -0.61 | Intra_Vcl_H5 | -0.52 | -0.92 |
| Intra_ke_Cl(out) | -1.49 | -0.64 | Intra_Vcl_Cl(out) | -0.79 | -0.26 |
| Intra_Vcl_Cl(in) | -1.91 | -0.99 | Intra_Vcl_C | -2.40 | -0.79 |
| Intra_ke_C | -1.94 | -0.99 | Intra_Vx_Cl(out) | -3.16 | -0.99 |
| Intra_Vx_Cl(out) | -4.71 | -0.97 | Intra_Vcl_Cl(in) | -9.25 | -0.98 |
| **X = Br** | | | | | |
| **Close Segment (ξ > -2.9)** | | | **Far Segment (ξ < -2.9)** | | |
| TERM | REG | R | TERM | REG | R |
| Intra_Vx_Br(in) | 5.60 | 0.96 | Intra_ke_Br(in) | 10.92 | 0.98 |
| Intra_Vcl_Br(out) | 3.18 | 0.98 | Intra_ke_Br(out) | 3.59 | 0.63 |
| Intra_Vcl_C | 1.85 | 0.98 | Intra_ke_C | 2.99 | 0.56 |
| Intra_Vx_C | 1.12 | 1.00 | Intra_Vx_Br(in) | 1.67 | 0.97 |
| Intra_ke_Br(out) | 0.35 | 0.23 | Intra_ke_H3 | 0.96 | 0.89 |
| Intra_Vx_H4 | 0.21 | 0.96 | Intra_Vx_C | 0.90 | 0.81 |
| Intra_Vx_H5 | 0.21 | 0.95 | Intra_ke_H5 | 0.80 | 0.87 |
| Intra_Vx_H3 | 0.20 | 0.95 | Intra_ke_H4 | 0.79 | 0.87 |
| Intra_ke_H3 | -0.03 | -0.26 | Intra_Vx_H5 | 0.12 | 0.57 |
| Intra_ke_H4 | -0.03 | -0.30 | Intra_Vx_H4 | 0.12 | 0.56 |
| Intra_ke_H5 | -0.03 | -0.30 | Intra_Vx_H3 | -0.02 | -0.09 |
| Intra_Vcl_H5 | -0.04 | -0.68 | Intra_Vcl_H4 | -0.39 | -0.85 |
| Intra_Vcl_H4 | -0.04 | -0.67 | Intra_Vcl_H5 | -0.40 | -0.85 |
| Intra_Vcl_H3 | -0.04 | -0.73 | Intra_Vcl_H3 | -0.51 | -0.89 |
| Intra_ke_Br(in) | -1.17 | -0.71 | Intra_Vcl_Br(out) | -0.99 | -0.23 |
| Intra_Vcl_Br(in) | -2.37 | -1.00 | Intra_Vcl_C | -2.03 | -0.52 |
| Intra_ke_C | -3.18 | -0.99 | Intra_Vx_Br(out) | -4.71 | -0.97 |
| Intra_Vx_Br(out) | -7.17 | -0.98 | Intra_Vcl_Br(in) | -9.24 | -0.99 |
| **X = I** | | | | | |
| **Close Segment (ξ > -3.3)** | | | **Far Segment (ξ < -3.3)** | | |
| TERM | REG | R | TERM | REG | R |
| Intra_Vx_I2 | 5.82 | 0.96 | Intra_ke_I2 | 11.34 | 0.99 |
| Intra_Vx_C | 2.37 | 0.98 | Intra_ke_I6 | 7.17 | 0.93 |
| Intra_Vcl_C | 1.82 | 0.96 | Intra_ke_C | 5.87 | 0.83 |
| Intra_ke_I6 | 1.82 | 0.71 | Intra_Vx_I2 | 1.65 | 0.98 |
| Intra_Vcl_I6 | 1.68 | 0.87 | Intra_ke_H4 | 0.95 | 0.95 |
| Intra_Vx_H3 | 0.27 | 0.98 | Intra_ke_H5 | 0.95 | 0.95 |
| Intra_Vx_H4 | 0.26 | 0.98 | Intra_ke_H3 | 0.74 | 0.91 |
| Intra_Vx_H5 | 0.25 | 0.98 | Intra_Vx_C | 0.55 | 0.42 |
| Intra_Vcl_H3 | -0.01 | -0.20 | Intra_Vx_H3 | 0.16 | 0.80 |
| Intra_Vcl_H5 | -0.02 | -0.52 | Intra_Vx_H5 | -0.01 | -0.08 |
| Intra_Vcl_H4 | -0.02 | -0.56 | Intra_Vx_H4 | -0.02 | -0.13 |
| Intra_ke_H4 | -0.10 | -0.76 | Intra_Vcl_H3 | -0.36 | -0.88 |
| Intra_ke_H5 | -0.10 | -0.76 | Intra_Vcl_H4 | -0.46 | -0.93 |
| Intra_ke_H3 | -0.12 | -0.79 | Intra_Vcl_H5 | -0.48 | -0.93 |
| Intra_Vcl_I2 | -1.28 | -0.93 | Intra_Vx_I6 | -4.18 | -0.94 |
| Intra_ke_I2 | -2.06 | -0.80 | Intra_Vcl_C | -4.86 | -0.91 |
| Intra_ke_C | -4.24 | -0.97 | Intra_Vcl_I6 | -5.10 | -0.90 |
| Intra_Vx_I6 | -8.75 | -0.99 | Intra_Vcl_I2 | -9.61 | -0.99 |

**Discussion of Table S4**

Table S4 shows the general trend that, when the reaction is far from the transition state (segment “far”), the intra-atomic energy contributions that most destabilise the transition state are the kinetic energies of the three non-hydrogen atoms (F_out_, F_in_ and C). Secondly, the REG value corresponding to the “Intra” V_cl_ energy of F_out_ is larger than that of the Intra V_xc_ of F_in_, when close to the transition state (“segment close”). This statement is in agreement with our observations that F is dominated by its electrostatic contributions. On the other hand, for Cl and Br, the exchange-correlation of the X_in_ atom is the most prevalent, with the electrostatic component of X_out_ being the second largest destabilising contribution. This statement is in agreement with our observations that Cl and Br are dominated by their exchange-correlation contributions although the electrostatic contribution still matters. Furthermore, for I, the intra V_xc_ of I_in_ is the most dominant contribution in destabilising the transition state (in a similar fashion to Cl and Br) but the intra-atomic V_cl_ of I_out_ is not a large contributor to the destabilisation of the transition state (because it does not have the second largest REG, while Cl and Br do). This observation is in agreement with our previous observations that the electrostatics decrease in importance in going from F to I.
